# Inference for a Kavya–Manoharan Inverse Length Biased Exponential Distribution under Progressive-Stress Model Based on Progressive Type-II Censoring

**DOI:** 10.3390/e24081033

**Published:** 2022-07-27

**Authors:** Naif Alotaibi, Atef F. Hashem, Ibrahim Elbatal, Salem A. Alyami, A. S. Al-Moisheer, Mohammed Elgarhy

**Affiliations:** 1 Department of Mathematics and Statistics, College of Science Imam Mohammad Ibn Saud Islamic University (IMSIU), Riyadh 11432, Saudi Arabia; affaragalla@imamu.edu.sa (A.F.H.); iielbatal@imamu.edu.sa (I.E.); saalyami@imamu.edu.sa (S.A.A.); 2 Mathematics and Computer Science Department, Faculty of Science, Beni-Suef University, Beni-Suef 62511, Egypt; 3 Department of Mathematics, College of Science, Jouf University, P.O. Box 848, Sakaka 72351, Saudi Arabia; asalmoisheer@ju.edu.sa; 4 The Higher Institute of Commercial Sciences, Al Mahalla Al Kubra 31951, Egypt; m_elgarhy85@sva.edu.eg

**Keywords:** progressive-stress model, progressive censoring, maximum likelihood estimation, maximum product spacing, Kavya–Manoharan class of distributions, inverse length biased exponential distribution

## Abstract

In this article, a new one parameter survival model is proposed using the Kavya–Manoharan (KM) transformation family and the inverse length biased exponential (ILBE) distribution. Statistical properties are obtained: quantiles, moments, incomplete moments and moment generating function. Different types of entropies such as Rényi entropy, Tsallis entropy, Havrda and Charvat entropy and Arimoto entropy are computed. Different measures of extropy such as extropy, cumulative residual extropy and the negative cumulative residual extropy are computed. When the lifetime of the item under use is assumed to follow the Kavya–Manoharan inverse length biased exponential (KMILBE) distribution, the progressive-stress accelerated life tests are considered. Some estimating approaches, such as the maximum likelihood, maximum product of spacing, least squares, and weighted least square estimations, are taken into account while using progressive type-II censoring. Furthermore, interval estimation is accomplished by determining the parameters’ approximate confidence intervals. The performance of the estimation approaches is investigated using Monte Carlo simulation. The relevance and flexibility of the model are demonstrated using two real datasets. The distribution is very flexible, and it outperforms many known distributions such as the inverse length biased, the inverse Lindley model, the Lindley, the inverse exponential, the sine inverse exponential and the sine inverse Rayleigh model.

## 1. Introduction

Accelerated life tests (ALTs) are applied to gain rapid information on the lifetime distribution of materials or products. In ALTs, the units’ test is performed at higher-than-normal levels of stress (voltage, vibration, pressure, temperature, etc.) to induce early failures. Data obtained at the accelerated conditions are analyzed in terms of an appropriate statistical model and then extrapolated to the specified normal stress to estimate the lifetime distribution in normal use conditions. There are different methods to apply the stress. Commonly used methods are *constant*-stress, *step*-stress and *progressive*-stress; see, for example, Nelson [1], AL-Hussaini and Abdel-Hamid [2,3], Abdel-Hamid and AL-Hussaini [4] and Abdel-Hamid and Hashem [5]. The stress applied to a test product increases in time during a progressive-stress ALT; see Yin and Sheng [6], Abdel-Hamid and AL-Hussaini [7], Abdel-Hamid and Abushal [8], AL-Hussaini et al. [9] and Nadarajah et al. [10].

**Figure 1:**
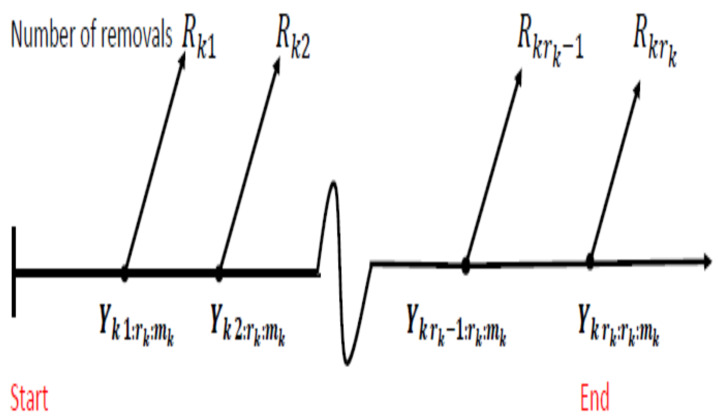
The process of generating order statistics under progressive type-II censoring.

Censoring has an important role in reliability and lifetime studies when the experimenter can not observe the lifetimes of all test units. Type-I and type-II censoring are two commonly used censoring schemes (CSs); see for example, Mann et al. [11], Meeker and Escobar [12] and Lawless [13]. Progressive type-II censoring, see Figure 1, is considered a generalization of type-II censoring. It allows the experimenter to remove units from a life test at different steps through the experiment. It saves time and cost that may be a consequence of such sampling scheme. For more details on progressive censoring, see Balakrishnan and Sandhu [14], Aggarwala and Balakrishnan [15], Balakrishnan and Aggarwala [16] and Hashem and Alyami [17].

In recent years, many various statisticians have been drawn to create families of distributions such as Marshall-Olkin-G [18], Kumaraswamy-G (Kum-G) in [19], odd Lomax-G [20], sine- G in [21], odd Dagum-G [22], Type II half logistic-G in [23], transmuted geometric-G [24], odd Perks- G in [25], odd Lindley- G in [26], truncated Cauchy power Weibull-G [27], generalized transmuted-G [28], truncated Cauchy power-G in [29], Burr X-G (BX-G) class [30], transmuted odd Fréchet-G in [31], Type II exponentiated half logistic– G in [32], Topp Leone-G in [33], exponentiated M-G by [34], odd Nadarajah–Haghighi-G in [35], exponentiated truncated inverse Weibull-G in [36] and T-X generator proposed in [37], among others.

Additional parameters give greater flexibility, but they also increase the complexity of estimation. To counter this, Ref. [38] proposed the Dinesh–Umesh–Sanjay (DUS) transformation to obtain new parsimonious classes of distributions. This is as follows. If G(x) is the baseline cumulative distribution function (CDF), the DUS transformation generates a new CDF F(x) expressed as:

$$F\left(x\right)=\frac{e^{G(x)}-1}{e-1},\;\;\;x\in R.$$

The merit of using this transformation is that the resulting distribution is parameter-parsimonious because no extra parameters are added. In this way, Ref. [39] proposed a new class of distributions that includes many flexible hazard rates. They explored using the DUS transformation using the exponentiated cdf, introducing the generalized DUS (GDUS) transformation. Ref. [40] proposed a generalized lifetime model based on the DUS transformation, with the CDF of the GDUS transformation given by

$$F\left(x;\alpha ,\zeta \right)=\frac{\exp\left(G^{\alpha }\left(x;\zeta \right)\right)-1}{e-1},\;\;x\in R,\;\;\;\alpha >0,$$

where α>0. The associated density function (PDF) is given by:

$$f\left(x;\alpha ,\zeta \right)=\frac{\alpha g\left(x;\zeta \right)G^{\alpha -1}\left(x;\zeta \right)\exp\left(G^{\alpha }\left(x;\zeta \right)\right)}{e-1},\;\;x\in R,\;\;\;\alpha >0,$$

where G(x;ζ) is the baseline distribution in the GDUS family distribution. This approach will always create a parsimonious distribution because it is a transformation rather than a generalization, so that no additional parameters beyond those in the baseline distribution are introduced.

Recently, Ref. [41] introduced a new transformation, the KM transformation family of distributions. The CDF and PDF are, respectively,

(1) $$F_{{}_{KM}}\left(x\right)=\frac{e}{e-1}\left(1-e^{-G(x)}\right),\;\;\;\;\;x\in R,$$

and

(2) $$f_{KM}\left(x\right)=\frac{e}{e-1}g\left(x\right)e^{-G(x)},\;\;\;\;\;x\in R.$$

The hazard rate function (HRF) is provided via

(3) $$\xi_{{}_{KM}}\left(x\right)=\frac{g\left(x\right)e^{1-G(x)}}{e^{1-G(x)}-1},\;\;\;x\in R.$$

Using a given baseline distribution, this family generates new lifetime models or distributions.

Ref. [41] used the exponential and Weibull distributions as baseline distributions because they are widely used in reliability theory and survival analysis.

Ref. [42] presented the length biased exponential (LBE) (or moment exponential (ME) model) by allocating weight to the exponential (E) model. They established that the LBE distribution is more adaptable than the E model. The CDF and PDF files are available:

(4) $$G\left(z;\theta \right)=1-\left(1+\frac{z}{\theta }\right)e^{-\frac{z}{\theta }},\;\;\;\;\;\;\;\;\;\;z>0,\;$$

and

(5) $$g\left(z;\theta \right)=\frac{z}{\theta^{2}}e^{-\frac{z}{\theta }},\;\;\;\;\;z>0,\;$$

respectively, where θ>0 is a scale parameter.

The inverse LBE (ILBE) distribution was presented in [43], and it is produced by utilizing the random variable X=1/Z, where *X* is as follows (5). The CDF and PDF files in the ILBE distribution are specified as

(6) $$G\left(x;\theta \right)=\left(1+\frac{\theta }{x}\right)e^{-\frac{\theta }{x}},\;\;\;\;\;\;\;\;\;\;x>0,\;\;\;\;\theta >0,\;$$

and

(7) $$g\left(x;\theta \right)=\frac{\theta^{2}}{x^{3}}e^{-\frac{\theta }{x}},\;\;\;\;\;\;\;\;\;x>0,\;\;\;\;\theta >0.\;$$

The fundamental goal of the article under consideration is to introduce the KMILBE model, as a new one-parameter lifetime model based on the KM transformation family, ILBE distribution, and also to investigate its statistical characteristics. The following points provide sufficient incentive to study the KMILBE distribution. We specify it as follows: (i) It is remarkable to observe the flexibility of the proposed model with the diverse graphical shapes of pdf and hrf. Thus, the the pdf of the KMILBE distribution can be unimodal and right-skewed, with very heavy tails, but the hrf of the KMILBE distribution can be increasing, J-shaped form; (ii) The KMILBE distribution have a closed form of the quantile function; (iii) The KMILBE is a good alternative to several lifetime distributions for modeling skewed data in applications; (iv) Different types of entropy and extropy are computed; (v) Based on progressive type-II censoring, we have discussed some estimation methods on a progressive-stress model when the lifetime of a product follows the KMILBE distribution. The methods that have been discussed are maximum likelihood (ML), least squares (LS), weighted least squares (WLS) and maximum product of spacing (MPS) estimation.

This paper is organized as follows: In Section 2, a new lifetime model using inverse length biased distribution as the baseline distribution in the KM transformation family is presented. In Section 3, we demonstrate the statistical features of the KMILBE model. Different measures of entropy are discussed in Section 4. In addition, some measures of extropy are proposed in Section 5. Model description and progressive type-II censoring by using ML, LS, WLS, and MPS are studied in Section 6. The simulation study and the numerical results are discussed in Section 7. Application to two real datasets is discussed in Section 8. Finally, concluding remarks are proposed in Section 9.

## 2. Construction of the Kavya–Manoharan Inverse Length Biased Exponential Distribution

In this section, we construct a new flexible distribution called the Kavya–Manoharan transformation inverse length biased exponential (KMILBE) distribution by inserting Equation (6) into Equation (1), to obtain

(8) $$F_{KMILBE}\left(x;\theta \right)=\frac{e}{e-1}\left\{1-e^{-\left(1+\frac{\theta }{x}\right)\;e^{-\frac{\theta }{x}}}\right\},\;\;\;\;\;\;\;\;\;x>0,\;\;\;\;\theta >0,$$

and the corresponding PDF is

(9) $$f_{KMILBE}\left(x;\theta \right)=\frac{e\;\theta^{2}}{e-1}x^{-3}e^{-\frac{\theta }{x}}e^{-\left(1+\frac{\theta }{x}\right)\;e^{-\frac{\theta }{x}}},\;\;\;\;\;\;\;\;\;x>0,\;\;\;\;\theta >0.$$

The survival function (SF), HRF, reversed HRF and cumulative HRF for the KMILBE distribution are

$$R_{KMILBE}\left(x;\theta \right)=1-\frac{e}{e-1}\left\{1-e^{-\left(1+\frac{\theta }{x}\right)\;e^{-\frac{\theta }{x}}}\right\},$$

$$h_{KMILBE}\left(x;\theta \right)=\frac{e\;\theta^{2}x^{-3}e^{-\frac{\theta }{x}}e^{-\left(1+\frac{\theta }{x}\right)\;e^{-\frac{\theta }{x}}}}{e-1-e\left\{1-e^{-\left(1+\frac{\theta }{x}\right)\;e^{-\frac{\theta }{x}}}\right\}},$$

$$\tau_{KMILBE}\left(x;\theta \right)=\frac{\theta^{2}x^{-3}e^{-\frac{\theta }{x}}e^{-\left(1+\frac{\theta }{x}\right)\;e^{-\frac{\theta }{x}}}}{1-e^{-\left(1+\frac{\theta }{x}\right)\;e^{-\frac{\theta }{x}}}},$$

and

$$H_{KMILBE}\left(x;\theta \right)=-ln\left(1-\frac{e}{e-1}\left\{1-e^{-\left(1+\frac{\theta }{x}\right)\;e^{-\frac{\theta }{x}}}\right\}\right)\;.$$

**Figure 2:**
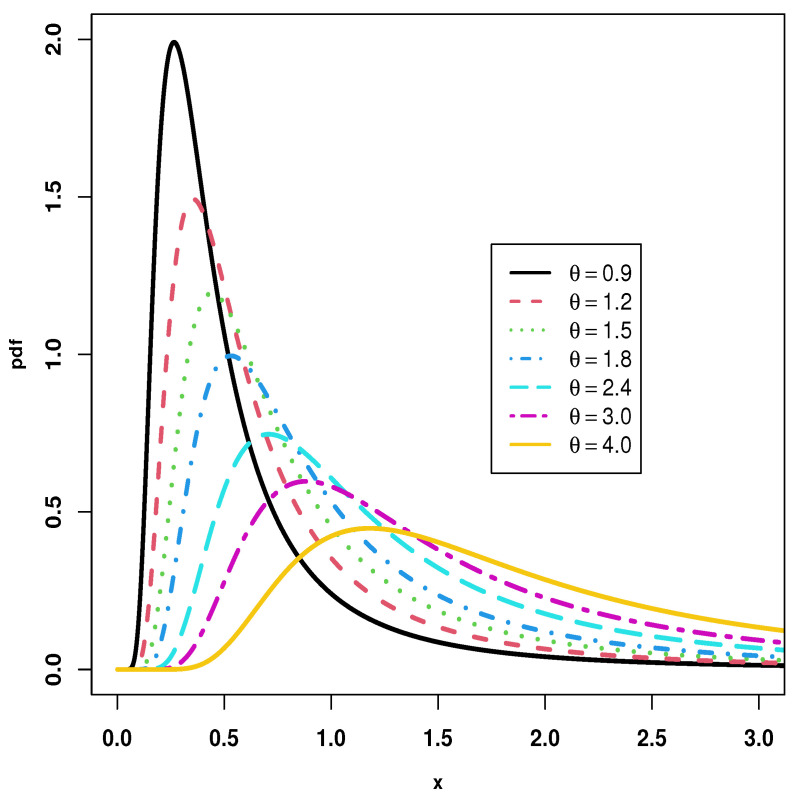
Different shapes of pdf for KMILBE distribution.

**Figure 3:**
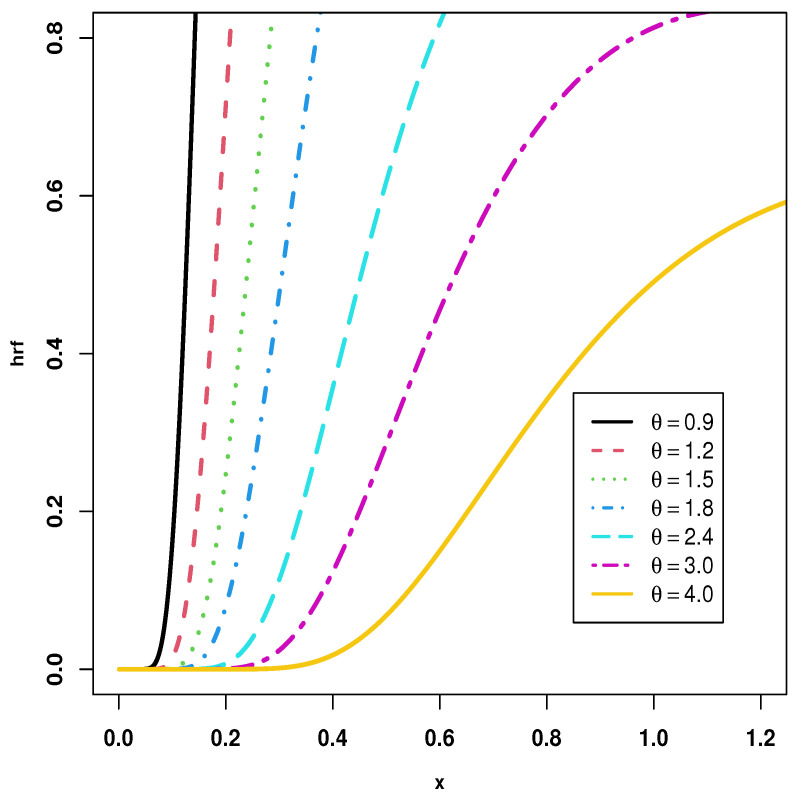
Different shapes of hrf for KMILBE distribution.

Figure 2 and Figure 3 show graphical representations of the PDF and the HRF of the KMILBE distribution with various values for the parameter θ. Forms of the PDF include right skewness and unimodal as shown in Figure 2. In addition, the forms of the HRF include increasing and J- shaped form, as shown in Figure 3. The KMILBE distribution is a very flexible model that provides different distributions when its parameters are changed.

## 3. Statistical Features of the New Suggested Model

This section provides the structural properties of the KMILBE, defined in Equation (9), including explicit expressions for quantile function (QF), linear representation of the density, *r*th ordinary and *s*th incomplete moments, and moment generating function.

### 3.1. Quantile Function

The QF, say $Q\left(u\right)=F^{-1}\left(u\right)$, u∈(0,1), is obtained by inverting Equation (8) as follows:

$$\frac{e}{e-1}\left\{1-e^{-\left(1+\frac{\theta }{Q(u)}\right)\;e^{-\frac{\theta }{Q(u)}}}\right\}=u,$$

which yields

$$\left(1+\frac{\theta }{Q(u)}\right)e^{-\frac{\theta }{Q(u)}}=-\ln\left[1-u\left(1-\frac{1}{e}\right)\right].$$

By multiplying the both sides by $e^{-1}$, then we have the Lambert equation

$$\left(1+\frac{\theta }{Q(u)}\right)e^{-\left(1+\frac{\theta }{Q(u)}\right)}=-e^{-1}\ln\left[1-u\left(1-\frac{1}{e}\right)\right].$$

Hence, we have the negative Lambert *W* function of the real argument

(10) $$Q_{u}=\frac{\theta }{-1-W_{-1}\left(-e^{-1}\ln\left[1-u\left(1-\frac{1}{e}\right)\right]\right)},$$

where u∈(0,1) and $W_{-1}\left(.\right)$ is the negative Lambert *W* function. By replacing u=0.5 in Equation (10), the median (Q2) of the KMILBE is readily available.

### 3.2. Useful Expansion

Here, we showed the useful expansion of the pdf, cdf and survival for the KMILBE distribution which can be used to drive several important properties of the KMILBE. According to the next exponential expansion

(11) $$e^{-\theta x}=\sum_{i=0}^{\infty }\frac{{\left(-1\right)}^{i}{\left(\theta x\right)}^{i}}{i!}.$$

By inserting the previous Equation (11) in Equation (9), we obtain

$$f_{KMILBE}\left(x;\theta \right)=\frac{e\theta^{2}}{e-1}x^{-3}\sum_{i=0}^{\infty }\frac{{\left(-1\right)}^{i}}{i!}{\left(1+\frac{\theta }{x}\right)}^{i}e^{-\frac{\left(i+1\right)\theta }{x}},$$

by applying the binomial expansion ${\left(1+z\right)}^{b}=\sum_{j=0}^{\infty }\left(\begin{array}{c} b\\ j \end{array}\right)z^{j},\;\;\;\;$ in the last equation, we can rewrite it as follows:

(12) $$f_{KMILBE}\left(x;\theta \right)=\sum_{i,j=0}^{\infty }\varpi_{i,j}x^{-j-3}e^{-\frac{\left(i+1\right)\theta }{x}},$$

where $\varpi_{i,j}=\frac{e}{e-1}\frac{\theta^{j+2}{\left(-1\right)}^{i}}{i!}\left(\begin{array}{c} i\\ j \end{array}\right)$.

In addition, we can obtain the expansion of $f_{KMILBE}^{\delta }\left(x;\theta \right)$ by using the last two expansions as follows:

(13) $$f_{KMILBE}^{\delta }\left(x;\theta \right)=\sum_{i,j=0}^{\infty }\eta_{i,j}x^{-j-3\delta }e^{-\frac{\left(i+\delta \right)\theta }{x}},$$

where, $\eta_{i,j}={\left(\frac{e\theta^{2}}{e-1}\right)}^{\delta }\frac{\theta^{j}{\left(-\delta \right)}^{i}}{i!}\left(\begin{array}{c} i\\ j \end{array}\right)$.

A gain using the previous expansions, then we can write the expansion of $R_{KMILBE}^{2}\left(x;\theta \right)$ as follows:

(14) $$R_{KMILBE}^{2}\left(x;\theta \right)=\sum_{i,j,k,m=0}^{\infty }\psi_{i,j,k,m}x^{-m}e^{-\frac{k\theta }{x}},$$

where $\psi_{i,j,k,m}={\left(\frac{e}{e-1}\right)}^{i}\frac{\theta^{m}j^{k}{\left(-1\right)}^{i+j+k}}{k!}\left(\begin{array}{c} 2\\ i \end{array}\right)$$\left(\begin{array}{c} i\\ j \end{array}\right)$$\left(\begin{array}{c} k\\ m \end{array}\right)$.

### 3.3. rth Moment

The *r*th ordinary or raw moments is an important measure to find measures of dispersion of the distribution. The following relationship is used to obtain the central or actual moments; the first moment about mean is always equal to zero, and the second moment about mean is equal to variance as $\mu_{2}=\mu_{2}^{\prime }-{\left(\mu_{1}^{\prime }\right)}^{2}$, $\mu_{3}=\mu_{3}^{\prime }-3\mu_{1}^{\prime }\mu_{2}^{\prime }+2{\left(\mu_{1}^{\prime }\right)}^{3}$ and $\mu_{4}=\mu_{4}^{\prime }-4\mu_{3}^{\prime }\mu_{1}^{\prime }+6\mu_{2}^{\prime }{\left(\mu_{1}^{\prime }\right)}^{2}-3{\left(\mu_{1}^{\prime }\right)}^{4}$. The moment based measure of skewness and kurtosis are obtained by using $\beta_{1}=\frac{\mu_{3}^{2}}{\mu_{2}^{3}}$ and $\beta_{2}=\frac{\mu_{4}}{\mu_{2}^{2}}$, respectively. Suppose that *X*∼ KMILBE (θ) for $x\in \left(0,\infty \right)$ and θ>0; then, its *r*th ordinary moment is given by

$$\mu_{r}^{\prime }=\sum_{i,j=0}^{\infty }\varpi_{i,j}\int_{0}^{\infty }x^{r-j-3}e^{-\frac{\left(i+1\right)\theta }{x}}dx.$$

Let $y=\frac{\left(i+1\right)\theta }{x}$; then,

$$\mu_{r}^{\prime }=\sum_{i,j=0}^{\infty }\varpi_{i,j}\int_{0}^{\infty }{\left[\left(i+1\right)\theta \right]}^{r-j-2}y^{j-r+1}e^{-y}dy,$$

(15) $$\mu_{r}^{\prime }=\sum_{i,j=0}^{\infty }\varpi_{i,j}{\left[\left(i+1\right)\theta \right]}^{r-j-2}\Gamma \left[j-r+2]\right.,\;\;\;\;\;j+2<r.$$

For *r* = 1, the mean of KMILBE is yielded as $\mu_{1}^{\prime }=\sum_{i,j=0}^{\infty }\varpi_{i,j}{\left[\left(i+1\right)\theta \right]}^{-j-1}\Gamma \left[j+1]\right.$.

### 3.4. Inverse rth Moment

Suppose that *X*∼ KMILBE (θ) for $x\in \left(0,\infty \right)$ and θ>0; then, its inverse *r*th moment is given by

$$\mu_{-r}^{\prime }=\sum_{i,j=0}^{\infty }\varpi_{i,j}\int_{0}^{\infty }x^{-r-j-3}e^{-\frac{\left(i+1\right)\theta }{x}}dx.$$

Let $y=\frac{\left(i+1\right)\theta }{x}$; then,

$$\mu_{-r}^{\prime }=\sum_{i,j=0}^{\infty }\varpi_{i,j}\int_{0}^{\infty }{\left[\left(i+1\right)\theta \right]}^{-r-j-2}y^{j+r+1}e^{-y}dy,$$

(16) $$\mu_{-r}^{\prime }=\sum_{i,j=0}^{\infty }\varpi_{i,j}{\left[\left(i+1\right)\theta \right]}^{-r-j-2}\Gamma \left[r+j+2]\right.$$

For *r* = 1, the harmonic mean of KMILBE is yielded as $\mu_{-1}^{\prime }=\sum_{i,j=0}^{\infty }\varpi_{i,j}{\left[\left(i+1\right)\theta \right]}^{-j-3}\Gamma \left[j+3]\right.$.

### 3.5. sth Incomplete Moment

The *s*th incomplete moment is an important measure and has wide applications in order to compute mean deviation from mean and median, mean waiting time, conditional moments and income inequality measures.

Suppose that *X*∼ KMILBE (θ) for $x\in \left(0,\infty \right)$ and θ>0; then, its *s*th incomplete moments by using (12) and lower incomplete gamma function $\gamma \left(a,t\right)=\int_{0}^{t}x^{a-1}e^{-x}dx$ are given by

(17) $$\varphi_{s}\left(w\right)=\sum_{i,j=0}^{\infty }\varpi_{i,j}{\left[\left(i+1\right)\theta \right]}^{r-j-2}\Gamma \left[j-r+2,\frac{(i+1)\theta }{w}\right],\;\;\;\;\;j+2<r.$$

### 3.6. Moment Generating Function

By definition, the moment generating function, $M\left(t\right)=E\left[e^{tX}\right]=\int e^{tx}f\left(x\right)dx$, can be yielded as Assume that *X*∼ KMILBE (θ) for $x\in \left(0,\infty \right)$ and θ>0; then, its moments generating function can be obtained by using (12) and replacing $e^{tx}=\sum_{r=0}^{\infty }\frac{t^{r}}{r!}x^{r}$ is given by

(18) $$E\left[e^{tX}\right]=\sum_{r=0}^{\infty }\sum_{i,j=0}^{\infty }\varpi_{i,j}\frac{t^{r}}{r!}{\left[\left(i+1\right)\theta \right]}^{r-j-2}\Gamma \left[j-r+2]\right.,$$

where j+2<r.

## 4. Entropy Measures

Entropy is a measure of a system’s variation, instability or unpredictability.

### 4.1. The Rényi Entropy

The Rényi entropy [44] is important in ecology and statistics as an index of diversity. For δ>0 and δ≠1, it is defined by the following expression:

(19) $$I_{\delta }\left(X\right)={\left(1-\delta \right)}^{-1}\log\int_{0}^{+\infty }f{\left(x\right)}^{\delta }dx\;.$$

By using Equation (13), we obtain

$$I_{\delta }\left(X\right)={\left(1-\delta \right)}^{-1}\log\left[\sum_{i,j=0}^{\infty }\eta_{i,j}{\left[\left(i+\delta \right)\theta \right]}^{1-j-3\delta }\Gamma \left[j+3\delta -1\right]\right],\;\;\;.$$

### 4.2. The Tsallis Entropy

The Tsallis entropy measure (see [45]) is defined by:

(20) $$T_{\delta }\left(X\right)=\frac{1}{\delta -1}\left[1-\int_{0}^{\infty }f^{\delta }\left(x\right)dx\right],\;\;\;\;\;\delta \neq 1,\;\;\;\delta >0.$$

By using Equation (13), we obtain

$$T_{\delta }\left(X\right)=\frac{1}{\delta -1}\left[1-\sum_{i,j=0}^{\infty }\eta_{i,j}{\left[\left(i+\delta \right)\theta \right]}^{1-j-3\delta }\Gamma \left[j+3\delta -1\right]\right].$$

### 4.3. The Havrda and Charvat Entropy

The Havrda and Charvat entropy measure (see [46]) is defined by:

(21) $$HC_{\delta }\left(X\right)=\frac{1}{2^{1-\delta }-1}\left[{\left(\int_{0}^{\infty }f^{\delta }\left(x\right)dx\right)}^{\frac{1}{\delta }}-1\right],\;\;\;\;\;\delta \neq 1,\;\;\;\delta >0.$$

By using Equation (13), we obtain

$$HC_{\delta }\left(X\right)=\frac{1}{2^{1-\delta }-1}\left[{\left(\sum_{i,j=0}^{\infty }\eta_{i,j}{\left[\left(i+\delta \right)\theta \right]}^{1-j-3\delta }\Gamma \left[j+3\delta -1\right]\right)}^{\frac{1}{\delta }}-1\right],\;\;\;\;\;\delta \neq 1,\;\;\;\delta >0.$$

### 4.4. The Arimoto Entropy

The Arimoto entropy measure (see [47]) is defined by:

(22) $$A_{\delta }\left(X\right)=\frac{\delta }{1-\delta -1}\left[{\left(\int_{0}^{\infty }f^{\delta }\left(x\right)dx\right)}^{\frac{1}{\delta }}\right],\;\;\;\;\;\delta \neq 1,\;\;\;\delta >0.$$

By using Equation (13), we obtain

$$A_{\delta }\left(X\right)=\frac{\delta }{1-\delta }\left[{\left(\sum_{i,j=0}^{\infty }\eta_{i,j}{\left[\left(i+\delta \right)\theta \right]}^{1-j-3\delta }\Gamma \left[j+3\delta -1\right]\right)}^{\frac{1}{\delta }}-1\right],\;\;\;\;\;\delta \neq 1,\;\;\;\delta >0.$$

## 5. Different Measures of Extropy

### 5.1. Extropy

Recently, an alternative measure of uncertainty, named by extropy was proposed by [48]. For an absolutely continuous non-negative random variable *X* with PDF *f* and CDF *F*, the extropy is defined as

(23) $$J\left(X\right)=-\frac{1}{2}\int_{0}^{+\infty }{\left[f(x)\right]}^{2}dx.$$

By using Equation (13), and putting δ=2, we obtain

$$J\left(X\right)=-\frac{1}{2}\left[\sum_{i,j=0}^{\infty }\eta_{i,j}{\left[\left(i+2\right)\theta \right]}^{-j-5}\Gamma \left[j+5\right]\right].$$

### 5.2. The Cumulative Residual Extropy

The cumulative residual extropy (CREX) was proposed by [49] analogous with (23) as a measure of uncertainty of random variables. The CREX is defined as

(24) $$J^{*}\left(X\right)=-\frac{1}{2}\int_{0}^{+\infty }R^{2}\left(x\right)dx.$$

It is always non-positive. By using Equation (14), we obtain

$$J^{*}\left(X\right)=-\frac{1}{2}\left[\sum_{i,j,k,m=0}^{\infty }\psi_{i,j,k,m}{\left[k\theta \right]}^{1-m}\Gamma \left[m-1\right]\right],\;\;\;\;m>1.$$

### 5.3. The Negative Cumulative Residual Extropy

Refs. [49,50] studied and investigated the negative CREX (NCREX) can be presented as

(25) $$J\left(X\right)=\frac{1}{2}\int_{0}^{+\infty }R^{2}\left(x\right)dx.$$

By using Equation (14), we obtain

$$J^{*}\left(X\right)=\frac{1}{2}\left[\sum_{i,j,k,m=0}^{\infty }\psi_{i,j,k,m}{\left[k\theta \right]}^{1-m}\Gamma \left[m-1\right]\right],\;\;\;\;m>1.$$

## 6. Model Description and Progressive Type-II Censoring

### 6.1. Cumulative Exposure Model

The cumulative exposure model (CEM) enables us to relate the distribution under progressive stress to the distribution under constant stress.

If the stress υ is a function of time *y*, υ=υ(y), and influences the scale parameter θ of the considered failure distribution, then θ becomes a function of *y*, θ(y) = θ(s(y)). Hence, the CEM takes the form; see Nelson [1],

(26) $$\Lambda \left(y\right)=\int_{0}^{y}\frac{dz}{\theta (\upsilon (z))}.$$

The CDF under progressive stress becomes

(27) G(y)=F(Λ(y)),

where F(.) is the assumed CDF with scale parameter equal to 1.

### 6.2. Basic Assumptions

1. First assumption: The relationship between the stress *s* and the scale parameter β satisfies the inverse power law i.e.,
  $$\theta \left(y\right)=\theta \left(\upsilon \left(y\right)\right)=\frac{1}{\eta \;{\left(\upsilon \left(y\right)\right)}^{\mu }},$$
  where υ is the applied stress and (η, μ) are two positive parameters to be estimated.
2. Second assumption: The stress υ(y) is a linearly increasing function in time *y*, i.e.,
  υ(y)=ωy,ω>0.
3. Third assumption: During the test process, the M units to be tested are divided into *ℓ*(>1) groups; each group includes $m_{k}$ units and is run under progressive stress. Thus,
  $$\upsilon_{k}=\omega_{k}y,\;k=1,\cdots ,\ell ,\;\omega_{1}<\omega_{2}<\cdots <\omega_{\ell }.$$
4. Fourth assumption: The failure times, denoted by $y_{k1}$, $y_{k2}$, ⋯, $y_{km_{k}}$, k=1,⋯,ℓ, are statistically independent.
5. Fifth assumption: The failure mechanisms of the failures are the same under any stress level.

From the first and second assumptions, the CEM (26) takes the form

(28) $$\Lambda_{k}\left(y\right)=\frac{\eta \;\omega_{k}^{\mu }\;y^{\mu +1}}{\mu +1},\;k=1,\cdots ,\ell .$$

From (8), CDF (27) under progressive stress takes the form

(29) $$G_{k}\left(y\right)\equiv G_{k}\left(y;\mu ,\eta \right)=\frac{e}{e-1}\left\{1-e^{-\left(1+\frac{\mu +1}{\eta \;\omega_{k}^{\mu }\;y^{\mu +1}}\right)\;e^{-\frac{\mu +1}{\eta \;\omega_{k}^{\mu }\;y^{\mu +1}}}}\right\}.$$

The corresponding PDF is given by

(30) $$g_{k}\left(y\right)\equiv g_{k}\left(y;\mu ,\eta \right)=\frac{e}{e-1}\;\frac{{\left(\mu +1\right)}^{3}}{\eta^{2}\;\omega_{k}^{2\mu }\;y^{2\mu +3}}\;e^{-\frac{\mu +1}{\eta \;\omega_{k}^{\mu }\;y^{\mu +1}}}\;e^{-\left(1+\frac{\mu +1}{\eta \;\omega_{k}^{\mu }\;y^{\mu +1}}\right)\;e^{-\frac{\mu +1}{\eta \;\omega_{k}^{\mu }\;y^{\mu +1}}}}.$$

### 6.3. Progressive Type-II Censoring

The progressive type-II censoring under progressive stress model can be applied as follows: Under Assumption 3, for k= 1, …, *ℓ*, suppose that $r_{k}$(<$m_{k}$) and $R_{k1}$, $R_{k2}$, …, $R_{kr_{k}}$ are fixed before the experiment. $R_{k1}$ surviving units are randomly removed from the test, when the first failure time in group *k* occurs and $R_{k2}$ surviving units are randomly removed from the test when the second failure time in group *k* occurs. The test continues in the same manner until the $r_{k}$-th failure at which all the remaining surviving units $R_{kr_{k}}$ = $m_{k}$ − $r_{k}$ − $\sum_{i=1}^{r_{k}-1}$$R_{ki}$ are removed from the test, thereby terminating the life-test.

The data from *ℓ* progressively type-II censored samples are as follows: ($y_{k1:r_{k}:m_{k}}$; $R_{k1}$), …, ($y_{kr_{k}:r_{k}:m_{k}}$; $R_{kr_{k}}$) where $y_{k1:r_{k}:m_{k}}$< … < $y_{kr_{k}:r_{k}:m_{k}}$ denote the $r_{k}$ ordered observed failure times, and $R_{k1}$, …, $R_{kr_{k}}$ denote the number of units removed from the experiment at failure times $y_{k1:r_{k}:m_{k}}$, …, $y_{kr_{k}:r_{k}:m_{k}}$.

Based on *ℓ* progressively type-II censored samples, under progressive stress ALT, the likelihood function is given by

(31) $$\begin{array}{cc} L(\mu ,\eta ;y)\propto & \prod_{k=1}^{\ell }\prod_{j=1}^{r_{k}}g_{k}\left(y_{kj}\right){\left[1-G_{k}\left(y_{kj}\right)\right]}^{R_{kj}}, \end{array}$$

where **y** = $\left(y_{1},y_{2},\cdots ,y_{\ell }\right)$, $y_{k}$ = $\left(y_{k1},\cdots ,y_{kr_{k}}\right)$, and $y_{kj}\equiv y_{kj:r_{k}:m_{k}},k=1,\cdots ,\ell ,j=1,\cdots ,r_{k}$,

Using Equations (29) and (30), the log-likelihood function takes the form

(32) $$\begin{array}{cc} \log[L(\mu ,\eta ;y)]\propto & 3D\log\left[\mu +1\right]-2D\log\left[\eta \right]-2\mu \sum_{k=1}^{\ell }r_{k}\log\left[\omega_{k}\right]-\left(2\mu +3\right)\sum_{k=1}^{\ell }\sum_{j=1}^{r_{k}}\log\left[y_{kj}\right]\\ \; & \;\;-\sum_{k=1}^{\ell }\sum_{j=1}^{r_{k}}\left(\varphi_{kj}+\left[1+\varphi_{kj}\right]e^{-\varphi_{kj}}\right)+\sum_{k=1}^{\ell }\sum_{j=1}^{r_{k}}R_{kj}\log\left[e^{1-\left(1+\varphi_{kj}\right)\;e^{-\varphi_{kj}}}-1\right], \end{array}$$

where $D=\sum_{k=1}^{\ell }r_{k}$ and

(33) $$\varphi_{kj}\equiv \varphi_{kj}\left(\mu ,\eta \right)=\frac{\mu +1}{\eta \;\omega_{k}^{\mu }\;y_{kj}^{\mu +1}}.$$

Then, the likelihood equations take the forms

(34) $$\begin{array}{cc} 0=\frac{\partial \log[L(\mu ,\eta ;y)]}{\partial \mu } & =\frac{3D}{\mu +1}-2\sum_{k=1}^{\ell }r_{k}\log\left[\omega_{k}\right]-2\sum_{k=1}^{\ell }\sum_{j=1}^{r_{k}}\log\left[y_{kj}\right]-\sum_{k=1}^{\ell }\sum_{j=1}^{r_{k}}A_{kj}\left[1-\varphi_{kj}\;e^{-\varphi_{kj}}\right]\\ \; & \;\;+\sum_{k=1}^{\ell }\sum_{j=1}^{r_{k}}R_{kj}\frac{A_{kj}\;\varphi_{kj}\;e^{-\varphi_{kj}}}{1-e^{-1+\left(1+\varphi_{kj}\right)\;e^{-\varphi_{kj}}}}, \end{array}$$

(35) $$\begin{array}{cc} 0=\frac{\partial \log[L(\mu ,\eta ;y)]}{\partial \eta } & =\frac{-2D}{\eta }-\sum_{k=1}^{\ell }\sum_{j=1}^{r_{k}}B_{kj}\left[1-\varphi_{kj}\;e^{-\varphi_{kj}}\right]+\sum_{k=1}^{\ell }\sum_{j=1}^{r_{k}}R_{kj}\frac{B_{kj}\;\varphi_{kj}\;e^{-\varphi_{kj}}}{1-e^{-1+\left(1+\varphi_{kj}\right)\;e^{-\varphi_{kj}}}}, \end{array}$$

where

(36) $$\begin{array}{cc} A_{kj}\equiv A_{kj}\left(\mu ,\eta \right) & =\frac{\partial \varphi_{kj}}{\partial \mu }=\frac{1-\left(\mu +1\right)\log\left[\omega_{k}\;y_{kj}\right]}{\eta \;\omega_{k}^{\mu }\;y_{kj}^{\mu +1}}, \end{array}$$

(37) $$\begin{array}{cc} B_{kj}\equiv B_{kj}\left(\mu ,\eta \right) & =\frac{\partial \varphi_{kj}}{\partial \eta }=\frac{-\mu -1}{\eta^{2}\;\omega_{k}^{\mu }\;y_{kj}^{\mu +1}}. \end{array}$$

The MLEs $\hat{\mu }$ and $\hat{\eta }$ of μ and η could be obtained by solving the likelihood equations, $\frac{\partial \log[L(\mu ,\eta ;y)]}{\partial \mu }=0$ and $\frac{\partial \log[L(\mu ,\eta ;y)]}{\partial \eta }=0$, with respect to μ and η and solving these equations simultaneously to obtain the MLEs. These equations can be numerically solved using iterative techniques using statistical software, since it is not possible for analytical solutions to obtain the roots.

Based on the common asymptotic normality theory of MLEs, we can consider that $\frac{\hat{\mu }-\mu }{\sqrt{\mathrm{Var}(\hat{\mu })}}$ and $\frac{\hat{\eta }-\eta }{\sqrt{\mathrm{Var}(\hat{\eta })}}$ can be approximated by a standard normal distribution, i.e.,

$$\frac{\hat{\mu }-\mu }{\sqrt{\mathrm{Var}(\hat{\mu })}}∼N\left(0,1\right)\;\mathrm{and}\;\frac{\hat{\eta }-\eta }{\sqrt{\mathrm{Var}(\hat{\eta })}}∼N\left(0,1\right),$$

where $\mathrm{Var}(\hat{\mu })$ and $\mathrm{Var}(\hat{\eta })$ are the variance of $\hat{\mu }$ and $\hat{\eta }$, which can be obtained from the inverse of the local Fisher information matrix (FIM),

(38) $$\begin{array}{cc} V=I^{-1}= & \left(\begin{array}{cc} \mathrm{Var}(\hat{\mu }) & \;\mathrm{Cov}(\hat{\mu },\hat{\eta })\\ \mathrm{Cov}(\hat{\mu },\hat{\eta }) & \;\mathrm{Var}(\hat{\eta }) \end{array}\right), \end{array}$$

where

(39) $$\begin{array}{cc} I=- & \left(\begin{array}{cc} \frac{\partial^{2}\hat{£}}{\partial \mu^{2}} & \;\frac{\partial^{2}\hat{£}}{\partial \mu \partial \eta }\\ \frac{\partial^{2}\hat{£}}{\partial \eta \partial \mu } & \;\frac{\partial^{2}\hat{£}}{\partial \eta^{2}} \end{array}\right), \end{array}$$

where the caret ^ denotes that the derivative is evaluated at $\left(\hat{\mu },\hat{\eta }\right)$. The second partial derivatives of the natural logarithm of the likelihood function with respect to μ and η can be obtained without difficulty.

Suppose that $\zeta_{1}=\mu$ and $\zeta_{2}=\eta$. Then, for i=1,2, a 100(1−ε)% normal approximation confidence interval (NACI) for $\zeta_{i}$ can be defined as

$$\left(\max\left\{0,\hat{\zeta_{i}}-z_{\varepsilon /2}\sqrt{\mathrm{Var}(\hat{\zeta_{i}})}\right\},\hat{\zeta_{i}}+z_{\varepsilon /2}\sqrt{\mathrm{Var}(\hat{\zeta_{i}})}\right),$$

where $\hat{\zeta_{i}}$ is the MLE of $\zeta_{i}$ and $z_{\varepsilon /2}$ is the upper ε/2 percentile of N(0,1) distribution.

Sometimes, the lower bound of NACI may have a negative value for the positive parameter. Thus, Meeker and Escobar [12] suggested using a log transformation confidence interval (LTCI) for this parameter. The normal approximation of log-transformed MLE, $\frac{\ln\hat{\zeta_{i}}-\ln\zeta_{i}}{\sqrt{\mathrm{Var}(\ln\hat{\zeta_{i}})}}$, i=1,2, can be approximated to a standard normal distribution i.e.,

$$\frac{\ln\hat{\zeta_{i}}-\ln\zeta_{i}}{\sqrt{\mathrm{Var}(\ln\hat{\zeta_{i}})}}∼N\left(0,1\right).$$

where $\mathrm{Var}\left(\ln\hat{\zeta_{i}}\right)=\frac{\mathrm{Var}(\hat{\zeta_{i}})}{{\hat{\zeta_{i}}}^{2}}$.

Therefore, a 100(1−ε)% LTCI for $\zeta_{i}$ can be defined as

$$\left(\hat{\zeta_{i}}\;\exp\left[-z_{\varepsilon /2}\frac{\sqrt{\mathrm{Var}(\hat{\zeta_{i}})}}{\hat{\zeta_{i}}}\right],\hat{\zeta_{i}}\;\exp\left[z_{\varepsilon /2}\frac{\sqrt{\mathrm{Var}(\hat{\zeta_{i}})}}{\hat{\zeta_{i}}}\right]\right).$$

### 6.4. Least Squares and Weighted Least Squares Estimations

The LS and WLS methods were introduced by Swain et al. [51] to estimate the Beta distribution parameters. Based on progressive type-II censoring, Abdel-Hamid and Hashem [52], and Hashem and Alyami [17], used these two methods to estimate the parameters included in the doubly Poisson-exponential and exponential-doubly Poisson distributions. They can be performed as follows: Let ($Y_{k1},\cdots ,Y_{kr_{k}}$), k=1,⋯,ℓ, be the ordered progressively type-II censored sample of size $r_{k}$ from the KMILBE distribution, under progressive stress ALT. The LS estimates (LSEs) of the unknown parameters can be obtained by minimizing the following quantity with respect to the unknown parameters: $\Psi \left(\mu ,\eta \right)=\sum_{k=1}^{\ell }\sum_{j=1}^{r_{k}}{\left(G_{k}\left(y_{kj}\right)-E\left[{\hat{G}}_{k}\left(y_{kj}\right)\right]\right)}^{2},$ where $E\left[{\hat{G}}_{k}\left(y_{kj}\right)\right]$ is the expectation of the empirical CDF, see Aggarwala and Balakrishnan [15], which is given by

$$\begin{array}{cc} E\left[{\hat{G}}_{k}\left(y_{kj}\right)\right] & =1-\prod_{s=r_{k}-j+1}^{r_{k}}\left[\frac{s+\sum_{i=r_{k}-s+1}^{r_{k}}R_{ki}}{1+s+\sum_{i=r_{k}-s+1}^{r_{k}}R_{ki}}\right],\;\;\;j=1,\cdots ,r_{k},\;k=1,\cdots ,\ell , \end{array}$$

Therefore, the LSEs $\overset{˘}{\mu }$ and $\overset{˘}{\eta }$ of μ and η can be obtained by minimizing the following quantity with respect to μ and η

$$\Psi \left(\mu ,\eta \right)=\sum_{k=1}^{\ell }\sum_{j=1}^{r_{k}}{\left(\frac{e}{e-1}\left\{1-e^{-\left(1+\varphi_{kj}\right)\;e^{-\varphi_{kj}}}\right\}-E\left[{\hat{G}}_{k}\left(y_{kj}\right)\right]\right)}^{2}.$$

These estimates can also be obtained by solving the nonlinear equations simultaneously to obtain the LSEs. These equations can be numerically solved using iterative techniques using statistical software since it is not possible for analytical solutions to obtain the roots:

(40) $$\begin{array}{cc} 0=\frac{\partial \Psi (\mu ,\eta )}{\partial \mu } & =\sum_{k=1}^{\ell }\sum_{j=1}^{r_{k}}\Upsilon_{kj}\;\left(\frac{e}{e-1}\left\{1-e^{-\left(1+\varphi_{kj}\right)\;e^{-\varphi_{kj}}}\right\}-E\left[{\hat{G}}_{k}\left(y_{kj}\right)\right]\right), \end{array}$$

(41) $$\begin{array}{cc} 0=\frac{\partial \Psi (\mu ,\eta )}{\partial \eta } & =\sum_{k=1}^{\ell }\sum_{j=1}^{r_{k}}\Omega_{kj}\;\left(\frac{e}{e-1}\left\{1-e^{-\left(1+\varphi_{kj}\right)\;e^{-\varphi_{kj}}}\right\}-E\left[{\hat{G}}_{k}\left(y_{kj}\right)\right]\right), \end{array}$$

where

(42) $$\begin{array}{cc} \Upsilon_{kj}\equiv \Upsilon_{kj}\left(\mu ,\eta \right) & =A_{kj}\;\varphi_{kj}\;e^{-\left[\varphi_{kj}+\left(1+\varphi_{kj}\right)\;e^{-\varphi_{kj}}\right]}, \end{array}$$

(43) $$\begin{array}{cc} \Omega_{kj}\equiv \Omega_{kj}\left(\mu ,\eta \right) & =B_{kj}\;\varphi_{kj}\;e^{-\left[\varphi_{kj}+\left(1+\varphi_{kj}\right)\;e^{-\varphi_{kj}}\right]}, \end{array}$$

and $\varphi_{kj},A_{kj}$ and $B_{kj}$ are given by (33), (36) and (37), respectively.

The WLS estimates (WLSEs) of the unknown parameters can be obtained by minimizing the following quantity with respect to the unknown parameters:

$$\Delta \left(\mu ,\eta \right)=\sum_{k=1}^{\ell }\sum_{j=1}^{r_{k}}\frac{1}{V[{\hat{G}}_{k}\left(y_{kj}\right)]}{\left(G_{k}\left(y_{kj}\right)-E\left[{\hat{G}}_{k}\left(y_{kj}\right)\right]\right)}^{2},$$

where $V\left[{\hat{G}}_{k}\left(y_{kj}\right)\right]$ is the variance of the empirical CDF, see Aggarwala and Balakrishnan [15], which is given by

$$\begin{array}{cc} V[{\hat{G}}_{k}\left(y_{kj}\right)] & =\left(\prod_{s=r_{k}-j+1}^{r_{k}}Q_{ks}\right)\left(\prod_{s=r_{k}-j+1}^{r_{k}}P_{ks}-\prod_{s=r_{k}-j+1}^{r_{k}}Q_{ks}\right),\;j=1,\cdots ,r_{k},\;k=1,\cdots ,\ell , \end{array}$$

where

$$\begin{array}{cc} P_{ks} & =Q_{ks}+\frac{1}{\left(1+s+\sum_{i=r_{k}-s+1}^{r_{k}}R_{ki}\right)\left(2+s+\sum_{i=r_{k}-s+1}^{r_{k}}R_{ki}\right)},\;s=1,\cdots ,r_{k},\;k=1,\cdots ,\ell ,\\ Q_{ks} & =\frac{s+\sum_{i=r_{k}-s+1}^{r_{k}}R_{ki}}{1+s+\sum_{i=r_{k}-s+1}^{r_{k}}R_{ki}},\;\;\;\;\;\;\;\;\;\;\;\;\;\;s=1,\cdots ,r_{k},\;k=1,\cdots ,\ell , \end{array}$$

The WLSEs $\tilde{\mu }$ and $\tilde{\eta }$ of μ and η can be obtained by minimizing the following quantity with respect to μ and η

$$\Delta \left(\mu ,\eta \right)=\sum_{k=1}^{\ell }\sum_{j=1}^{r_{k}}\frac{1}{V[{\hat{G}}_{k}\left(y_{kj}\right)]}{\left(\frac{e}{e-1}\left\{1-e^{-\left(1+\varphi_{kj}\right)\;e^{-\varphi_{kj}}}\right\}-E\left[{\hat{G}}_{k}\left(y_{kj}\right)\right]\right)}^{2}.$$

These estimates can also be obtained by solving the nonlinear equations simultaneously to obtain the WLSEs. These equations can be numerically solved using iterative techniques using statistical software since it is not possible for analytical solutions to obtain the roots:

(44) $$\begin{array}{cc} 0=\frac{\partial \Delta (\mu ,\eta )}{\partial \mu } & =\sum_{k=1}^{\ell }\sum_{j=1}^{r_{k}}\frac{\Upsilon_{kj}}{V[{\hat{G}}_{k}\left(y_{kj}\right)]}\;\left(\frac{e}{e-1}\left\{1-e^{-\left(1+\varphi_{kj}\right)\;e^{-\varphi_{kj}}}\right\}-E\left[{\hat{G}}_{k}\left(y_{kj}\right)\right]\right), \end{array}$$

(45) $$\begin{array}{cc} 0=\frac{\partial \Delta (\mu ,\eta )}{\partial \eta } & =\sum_{k=1}^{\ell }\sum_{j=1}^{r_{k}}\frac{\Omega_{kj}}{V[{\hat{G}}_{k}\left(y_{kj}\right)]}\;\left(\frac{e}{e-1}\left\{1-e^{-\left(1+\varphi_{kj}\right)\;e^{-\varphi_{kj}}}\right\}-E\left[{\hat{G}}_{k}\left(y_{kj}\right)\right]\right), \end{array}$$

where $\Upsilon_{kj}$ and $\Omega_{kj}$ are given by (42) and (43), respectively.

### 6.5. Maximum Product of Spacing Estimation

Cheng and Amin [53] introduced an alternative method to the ML method for estimating the unknown parameters in univariate continuous distributions. Based on progressive type-II censoring, Ng et al. [54] used this method to estimate the parameters included in the Weibull distribution. The Maximum product of spacing estimates (MPSEs) of the unknown parameters can be obtained by maximizing the following product of spacing with respect to the unknown parameters:

(46) $$\begin{array}{cc} S(\mu ,\eta ;y) & =\prod_{k=1}^{\ell }\left(\prod_{j=1}^{r_{k}+1}\left[G_{k}\left(y_{kj}\right)-G_{k}\left(y_{kj-1}\right)\right]\prod_{j=1}^{r_{k}}{\left[1-G_{k}\left(y_{kj}\right)\right]}^{R_{kj}}\right), \end{array}$$

where $G_{k}\left(y_{k0}\right)=0$ and $G_{k}\left(y_{kr_{k}+1}\right)=1$.

Using (29), the MPSEs $\tilde{\mu }$ and $\tilde{\eta }$ of μ and η can be obtained by maximizing the following product of spacing with respect to the μ and η

(47) $$\begin{array}{cc} S(\mu ,\eta ;y) & =\prod_{k=1}^{\ell }\left(\prod_{j=1}^{r_{k}+1}\frac{e}{e-1}\left[e^{-\left(1+\varphi_{kj-1}\right)\;e^{-\varphi_{kj-1}}}-e^{-\left(1+\varphi_{kj}\right)\;e^{-\varphi_{kj}}}\right]\right.\\ \; & \;\;\;\;\times \left.\prod_{j=1}^{r_{k}}{\left[\frac{e^{1-\left(1+\varphi_{kj}\right)\;e^{-\varphi_{kj}}}-1}{e-1}\right]}^{R_{kj}}\right), \end{array}$$

These estimates can also be obtained by solving the nonlinear equations simultaneously to obtain the MPSEs. These equations can be numerically solved using iterative techniques using statistical software since it is not possible for analytical solutions to obtain the roots:

(48) $$\begin{array}{cc} 0=\frac{\partial \log[S(\mu ,\eta )]}{\partial \mu } & =\sum_{k=1}^{\ell }\left(\sum_{j=1}^{r_{k}+1}\frac{\Upsilon_{kj-1}-\Upsilon_{kj}}{e^{-\left(1+\varphi_{kj-1}\right)\;e^{-\varphi_{kj-1}}}-e^{-\left(1+\varphi_{kj}\right)\;e^{-\varphi_{kj}}}}-\sum_{j=1}^{r_{k}}\frac{R_{kj}\Upsilon_{kj}}{e^{-\left(1+\varphi_{kj}\right)\;e^{-\varphi_{kj}}}-e^{-1}}\right),\; \end{array}$$

(49) $$\begin{array}{cc} 0=\frac{\partial \log[S(\mu ,\eta )]}{\partial \eta } & =\sum_{k=1}^{\ell }\left(\sum_{j=1}^{r_{k}+1}\frac{\Omega_{kj-1}-\Omega_{kj}}{e^{-\left(1+\varphi_{kj-1}\right)\;e^{-\varphi_{kj-1}}}-e^{-\left(1+\varphi_{kj}\right)\;e^{-\varphi_{kj}}}}-\sum_{j=1}^{r_{k}}\frac{R_{kj}\Omega_{kj}}{e^{-\left(1+\varphi_{kj}\right)\;e^{-\varphi_{kj}}}-e^{-1}}\right),\; \end{array}$$

where $\Upsilon_{kj}$ and $\Omega_{kj}$ are given by (42) and (43), respectively.

## 7. Simulation Study

As it is theoretically difficult to assess the efficiency of estimation methods, a Monte Carlo simulation is used to overcome this difficulty. In the current section, through Monte Carlo simulation, we conduct a numerical study to assess the efficiency and performance of the estimation methods according to the following steps:

1. Assign the values of $m_{k},r_{k}\left(1<r_{k}<m_{k}\right)$ and $(R_{kj},\cdots ,R_{kr_{k}}),k=1,\cdots ,\ell$.
2. For k=1,⋯,ℓ, generate a progressively type-II censored sample of size $r_{k}$ from the KMILBE distribution with CDF (29), according to the algorithm given in Balakrishnan and Sandhu [14].
3. The MLEs, MPSEs, LSEs, WLSEs, NACIs and LTCIs of the parameters μ and η are computed as shown in Section 2.
4. Evaluate the 95% NACIs and LTCIs of the parameters μ and η.
5. Repeat the above steps ℏ(=5000) times.
6. If $\hat{\beta }$ is an estimate of β, then the average of estimates, mean squared error (MSE) and relative absolute bias (RAB) of $\hat{\beta }$ over *ℏ* samples are given, respectively, by
  $$\begin{array}{c} \bar{\hat{\beta }}=\frac{1}{\hbar }\sum_{i=1}^{\hbar }{\hat{\beta }}_{i},\;\mathrm{MSE}\left(\hat{\beta }\right)=\frac{1}{\hbar }\sum_{i=1}^{\hbar }{\left({\hat{\beta }}_{i}-\beta \right)}^{2},\;\mathrm{RAB}\left(\hat{\beta }\right)=\frac{1}{\hbar }\sum_{i=1}^{\hbar }\frac{|{\hat{\beta }}_{i}-\beta |}{\beta }. \end{array}$$
7. Calculate the average of estimates of the parameters μ and η and their MSEs and RABs as shown in Step 5. Calculate also the mean of the MSEs (MMSE) and mean of the RABs (MRAB) according to the following two relations:
  $$\begin{array}{cc} \mathrm{MMSE} & =\frac{\mathrm{MSE}\left(\hat{\mu }\right)+\mathrm{MSE}\left(\hat{\eta }\right)}{2},\;\mathrm{MRAB}=\frac{\mathrm{RAB}\left(\hat{\mu }\right)+\mathrm{RAB}\left(\hat{\eta }\right)}{2}. \end{array}$$
8. Calculate the average interval lengths (AILs) and coverage probability (COVP) of the parameters μ and η.

The following three CSs are considered in the generation of samples:

- CS1: For k=1,…,ℓ
  $$\begin{array}{cc} R_{kj} & =m_{k}-r_{k},\;\;j=1,\\ R_{kj} & =0,\;\;\;\;\;\mathrm{otherwise}. \end{array}$$
- CS2: For k=1,…,ℓ
  $$\begin{array}{cc} R_{kj} & =m_{k}-r_{k},\;\;j=r_{k}/2\;\left(r_{k}\;\mathrm{is}\;\mathrm{even}\right),\mathrm{or}j=r_{k}+1/2\;\left(r_{k}\;\mathrm{is}\;\mathrm{odd}\right),\\ R_{kj} & =0,\;\;\;\;\;\mathrm{otherwise}. \end{array}$$
- CS3: For k=1,…,ℓ
  $$\begin{array}{cc} R_{kj} & =m_{k}-r_{k},\;\;j=r_{k},\\ R_{kj} & =0,\;\;\;\;\;\mathrm{otherwise}. \end{array}$$

The computational results are presented in Table 1, Table 2 and Table 3 taking into account the population parameter values: μ=0.2 and η=1.5. For the sake of comparison among the MLEs, MPSEs, LSEs, WLSEs, NACIs and LTCIs of the parameters μ and η, the total number of observations M is divided into two groups, ℓ=2, and another time into three groups, ℓ=3.

- In the case of two groups (ℓ=2), we consider
  $$\begin{array}{cc} m_{1} & =m_{2}=M/2,\\ r_{1} & =r_{2}=50\%,\;\;75\%\;\;\mathrm{and}\;\;100\%\;\;\mathrm{of}\;\mathrm{the}\;\mathrm{sample}\;\mathrm{size},\\ \omega_{1} & =1\;\;\mathrm{and}\;\;\omega_{2}=8. \end{array}$$
- In the case of three groups (ℓ=3), we consider
  $$\begin{array}{cc} m_{1} & =m_{2}=m_{3}=M/3,\\ r_{1} & =r_{2}=r_{2}=50\%,\;\;75\%\;\;\mathrm{and}\;\;100\%\;\;\mathrm{of}\;\mathrm{the}\;\mathrm{sample}\;\mathrm{size},\\ \omega_{1} & =1\;\;\omega_{2}=8,\;\;\mathrm{and}\;\;\omega_{3}=15. \end{array}$$

### Numerical Results

From Table 1, Table 2 and Table 3, we observe the following:

1. The MLEs are better than the LSEs and WLSEs through the AMSEs and ARABs;
2. The MLEs are better than the MSPEs through the AMSEs and ARABs for the parameter μ;
3. The WLSEs are better than the LSEs through the AMSEs and ARABs;
4. The MPSEs are better than the LSEs and WLSEs through the AMSEs;
5. The NACLs are better than the LTCIs via the AILs and COVP;
6. For ℓ=2,3, and fixed values of the total number of items to be tested, M, and hence fixed sample sizes, $m_{k}$, by increasing the failure times, $r_{k}$, the MSEs, AMSEs, RABs, ARABs and AILs of the considered parameters decrease.
7. For ℓ=2,3, and fixed values of the failure times, $r_{k}$ (=50%, 75% and 100% of the sample size $m_{k}$), by increasing the total number of items to be tested, M, the MSEs, AMSEs, RABs, ARABs and AILs of the considered parameters decrease.
8. For fixing the total number of items to be tested, by increasing *ℓ*, the MSEs, AMSEs, RABs and ARABs decrease.
9. By increasing the sample and failure time sizes ($r_{k}$, $m_{k}$), the COVP are close to 95%.
10. For fixed values of the sample and failure time sizes ($r_{k}$, $m_{k}$), the third CS gives more accurate results through the MSEs, AMSEs, RABs, ARABs and AILs than the other two CSs.

The above results are satisfied except for some rare cases; this may be due to fluctuation in the data.

## 8. Real Data Analysis

In this section, we illustrate the importance of the newly KMILBE distribution by utilizing two real-life datasets. We shall compare the fits of the KMILBE distribution with the following competing continuous distributions, which are reported in Table 4.

The fitted distributions are compared using the negative maximum log-likelihood (-LL), Akaike information criterion (AIC), corrected AIC (CAIC), Bayesian information criterion (BIC), Hannan Quinn information criterion Kolmogorov–Smirnov test (KS) and *p*-value (PV).

The first data set we consider in this paper is taken from [55]: 1501.82, 6989.43, 2424.02, 4150.29, 8693.35, 2643.77, 13,148.37, 6149.39, 23,587.21, 7248.37, 4788.22, 6009.51, 5349.65, 5741.32, 7065.81, 7261.37, 2358.42, 10,357.88, 2499.05, 3022.90, 4234.86, 4482.03, 6363.71, 3329.91, 8740.47, 3664.95, 4515.97, 8497.71, 4569.89, 8069.63, 7366.79, 1525.41, 3363.02, 2420.57, 3576.74, 3708.05, 5819.12, 5479.38. These data are carbon retained by leaves measured in kilogram/hectare for thirty-eight different plots of mountainous regions of Navarra (Spain), depending on the forest classification: areas with ninety percent or more beech trees (Fagus Sylvatica) are labeled monospecific, while areas with many species of trees are labeled multi specific.

The second data set: we consider data of times to infection of kidney dialysis patients in months, as described by [56]. The “times of infection” data set is: 2.5, 2.5, 3.5, 3.5, 3.5, 4.5, 5.5, 6.5, 6.5, 7.5, 7.5, 7.5,7.5, 8.5, 9.5, 10.5, 11.5, 12.5, 12.5, 13.5, 14.5, 14.5, 21.5, 21.5, 22.5, 22.5, 25.5, 27.5. Now, we make a normalization operation by divided these data by 30, to obtain data between 0 and1. The transformed data set becomes: 0.08333333, 0.08333333, 0.11666667, 0.11666667, 0.11666667, 0.15000000, 0.18333333,0.21666667, 0.21666667, 0.25000000, 0.25000000, 0.25000000, 0.25000000, 0.28333333, 0.31666667,0.35000000, 0.38333333, 0.41666667, 0.41666667, 0.45000000, 0.48333333, 0.48333333, 0.71666667,0.71666667, 0.75000000, 0.75000000, 0.85000000, 0.91666667.

**Figure 4:**
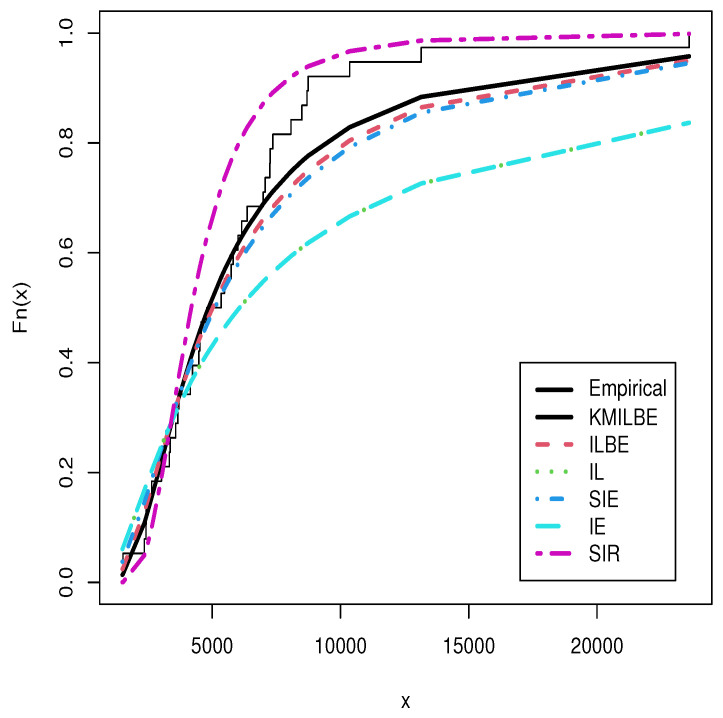
The fitted cdf plots for the data set 1.

**Figure 5:**
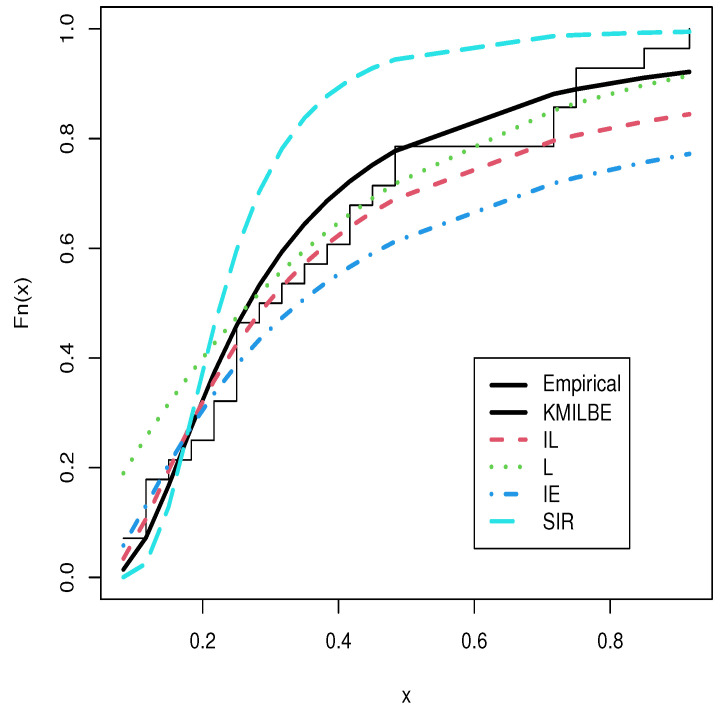
The fitted cdf plots for data set 2.

**Figure 6:**
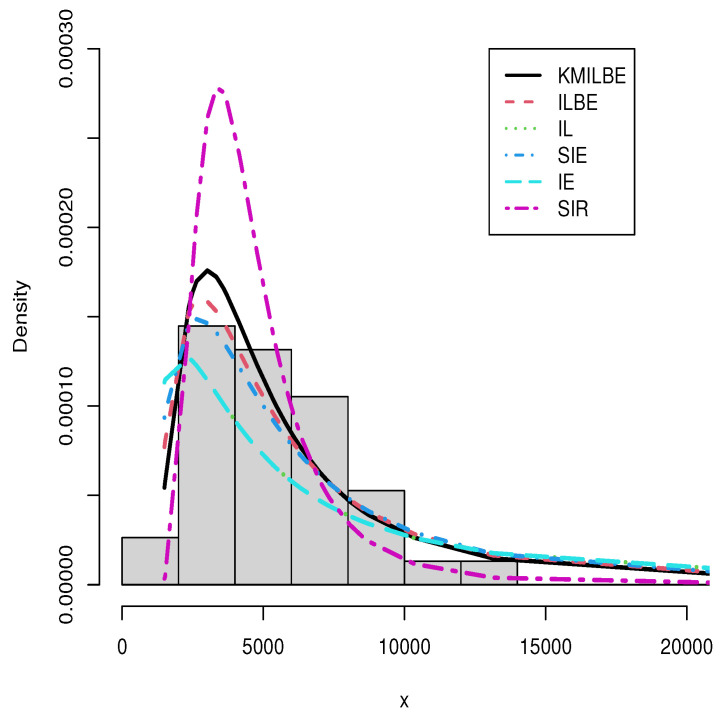
The fitted pdf plots for the data set 1.

**Figure 7:**
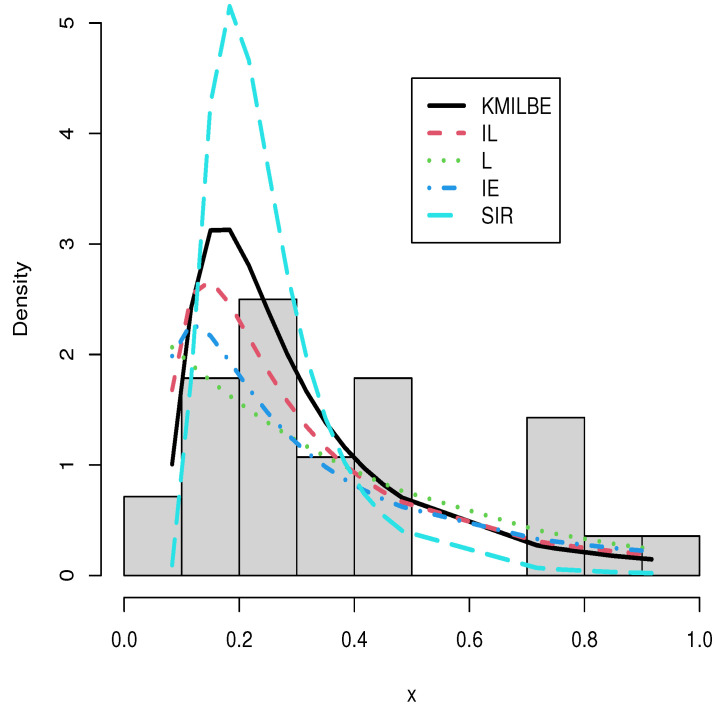
The fitted pdf plots for data set 2.

**Figure 8:**
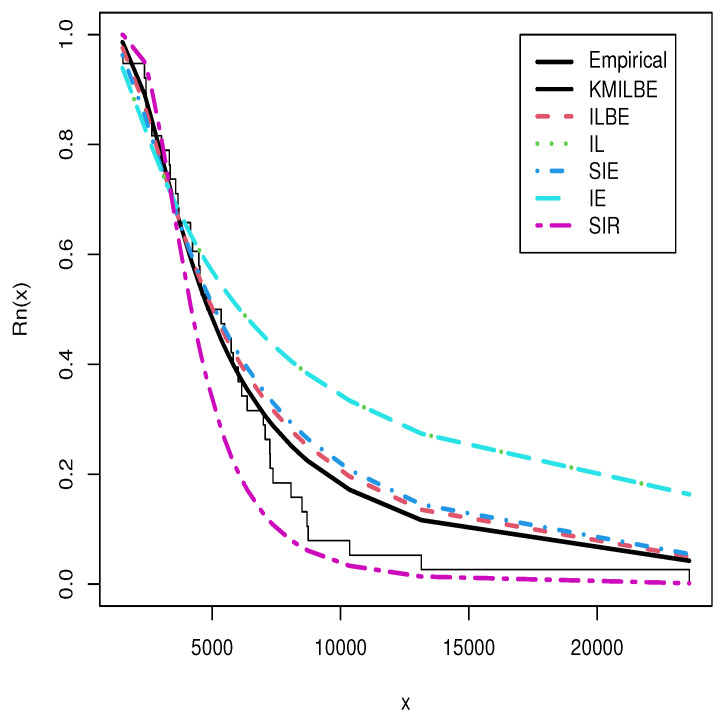
The fitted sf plots for data set 1.

**Figure 9:**
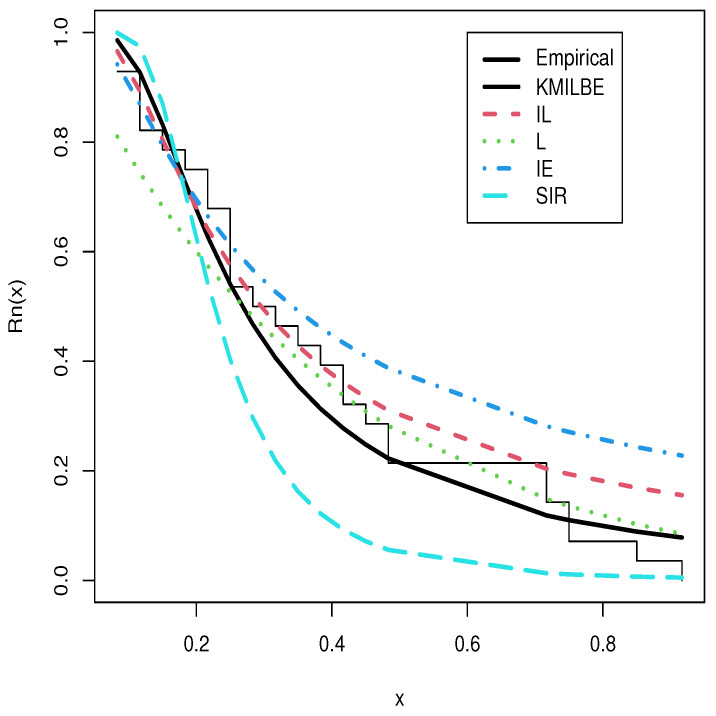
The fitted sf plots for data set 2.

**Figure 10:**
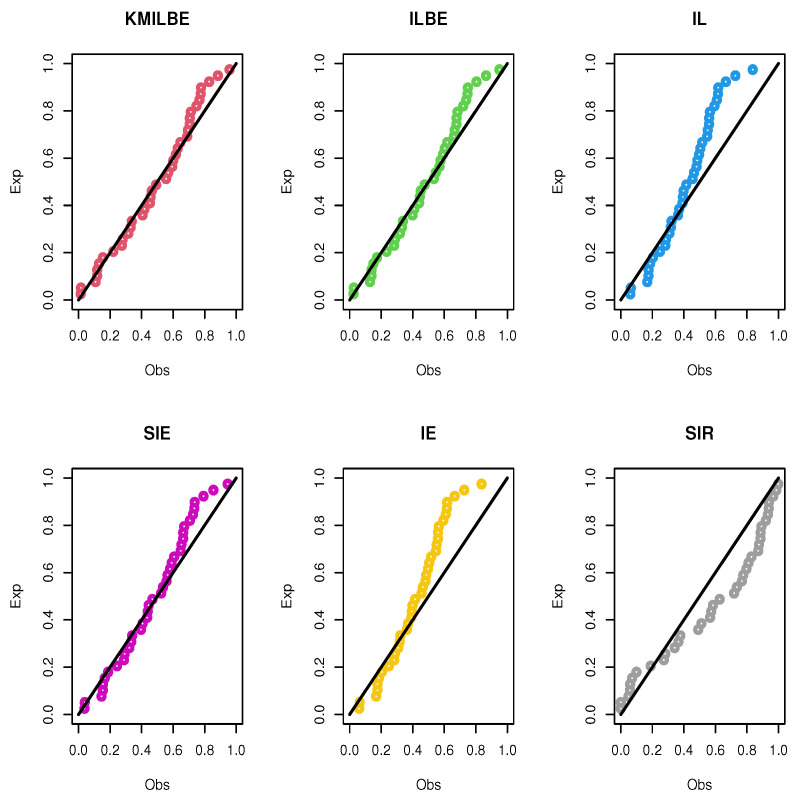
The P-P plots of the competing continuous models for data set 1.

**Figure 11:**
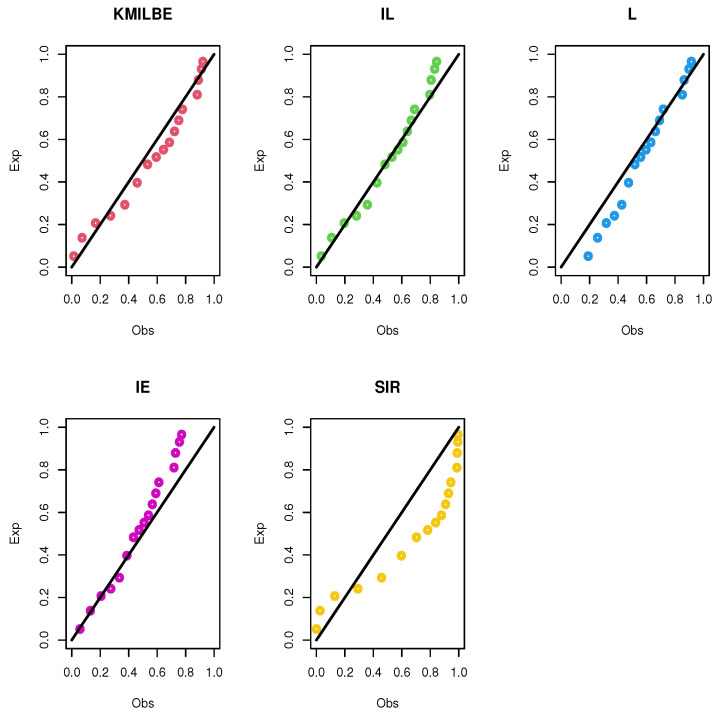
The P-P plots of the competing continuous models for data set 2.

The MLEs of the competing continuous models, standard errors (SEs), and goodness-of-fit measures are listed in Table 5 and Table 6 for the both datasets, respectively. For visual comparisons, the fitted CDF of the competitive distributions are depicted in Figure 4 and Figure 5, the fitted PDF of the competitive distributions are depicted in Figure 6 and Figure 7, the fitted sf of the competitive distributions are depicted in Figure 8 and Figure 9 respectively. Furthermore, P-P (probability–probability) plots of fitted distributions are displayed in Figure 10 and Figure 11 for the analyzed datasets, respectively. The findings in Table 5 and Table 6 illustrate that the KMILBE model provides a superior fit over other competing continuous models, since it has the lowest values for all measures and lowest value of the Kolmogorov–Smirnov distance (KS).

## 9. Conclusions

In this study, we explore a new one parameter model, which is called a Kavya–Manoharan inverse length biased exponential model. Its statistical and mathematical features (quantile, moments, inverse moments, incomplete moments and moment generating function) are derived. Different types of entropies such as Rényi entropy, Tsallis entropy, Havrda and Charvat entropy and Arimoto entropy are computed. Different measures of extropy such as extropy, cumulative residual extropy and the negative cumulative residual extropy are computed. Based on progressive type-II censoring, we have discussed some estimation methods on the progressive-stress model when the lifetime of a product follows the Kavya–Manoharan inverse length biased exponential distribution. The methods that have been discussed are ML, MPS, LS and WLS estimations. The approximate CIs for the unknown parameters have been established. The performance of these methods has been investigated through a simulation study, based on three different progressive CSs. The relevance and flexibility of the KMILBE model are demonstrated using two real datasets. 

## Figures and Tables

**Table 1:** MLEs and MPSEs of η and μ with their MSEs, RABs, AMSE and ARAB based on 5000 simulations. Population parameter values are η=1.5 and μ=0.2.

		$m_{1}$	$r_{1}$		MLE				MPSE			
		⋮	⋮		$\bar{\hat{\eta }}$	MSE($\hat{\eta }$)	RAB($\hat{\eta }$)	AMSE	$\bar{\overset{ˇ}{\eta }}$	MSE($\overset{ˇ}{\eta }$)	RAB($\overset{ˇ}{\eta }$)	AMSE
M	*ℓ*	$m_{\ell }$	$r_{\ell }$	CS	$\bar{\hat{\mu }}$	MSE($\hat{\mu }$)	RAB($\hat{\mu }$)	ARAB	$\bar{\overset{ˇ}{\mu }}$	MSE($\overset{ˇ}{\mu }$)	RAB($\overset{ˇ}{\mu }$)	ARAB
60	2	30	15	I	1.53024	0.05812	0.12577	0.03379	1.48487	0.04614	0.11121	0.02773
		30	15		0.22606	0.00947	0.37923	0.25250	0.16227	0.00932	0.40166	0.25644
				II	1.53595	0.04652	0.11181	0.02743	1.48572	0.0377	0.10132	0.02280
					0.22546	0.00834	0.35354	0.23268	0.17043	0.0079	0.36371	0.23251
				III	1.52858	0.04054	0.10481	0.02420	1.50566	0.03521	0.09698	0.02144
					0.22350	0.00785	0.34484	0.22482	0.18206	0.00768	0.35259	0.22478
			22	I	1.52152	0.04256	0.10759	0.02494	1.47824	0.03525	0.09856	0.02143
			22		0.22143	0.00731	0.33167	0.21963	0.16431	0.00761	0.35809	0.22832
				II	1.52260	0.03679	0.10087	0.02184	1.48067	0.03044	0.09125	0.01886
					0.22222	0.00688	0.32683	0.21385	0.16661	0.00729	0.34808	0.21966
				III	1.52217	0.03461	0.09761	0.02055	1.50056	0.02943	0.08901	0.01808
					0.22000	0.00649	0.31635	0.20698	0.17947	0.00672	0.33085	0.20993
			30	−−	1.5168	0.03236	0.09332	0.01928	1.47735	0.02745	0.08799	0.01709
			30		0.21873	0.0062	0.30563	0.19948	0.16573	0.00672	0.33395	0.21097
	3	20	10	I	1.50363	0.03188	0.09438	0.01999	1.51203	0.02856	0.08792	0.01866
		20	10		0.22289	0.00810	0.35086	0.22262	0.15476	0.00875	0.38819	0.23806
		20	10	2	1.50703	0.02563	0.08418	0.01633	1.50139	0.02327	0.07961	0.01546
					0.22049	0.00704	0.32220	0.20319	0.16055	0.00765	0.35851	0.21906
				III	1.50395	0.02286	0.08027	0.01485	1.51370	0.02116	0.07502	0.01396
					0.22105	0.00685	0.32158	0.20093	0.17491	0.00675	0.33257	0.20380
			15	I	1.49880	0.02440	0.08310	0.01544	1.50236	0.02143	0.07688	0.01451
			15		0.21841	0.00648	0.31646	0.19978	0.15325	0.00758	0.35827	0.21757
			15	2	1.49920	0.02138	0.07757	0.01355	1.50050	0.01861	0.07160	0.01273
					0.21803	0.00572	0.29586	0.18671	0.16032	0.00685	0.33739	0.20449
				III	1.49764	0.02039	0.07561	0.01307	1.51345	0.01954	0.07235	0.01301
					0.21837	0.00575	0.29681	0.18621	0.17124	0.00649	0.32721	0.19978
			20	−−	1.49669	0.01886	0.07293	0.01200	1.50042	0.01721	0.06919	0.01179
			20		0.21724	0.00514	0.28210	0.17752	0.15833	0.00638	0.32664	0.19791
			20									
120	2	60	30	I	1.51388	0.02722	0.08678	0.01597	1.47963	0.02448	0.08100	0.01534
		60	30		0.21869	0.00471	0.26997	0.17837	0.16768	0.00619	0.31628	0.19864
				II	1.51637	0.02148	0.07706	0.01271	1.48762	0.01830	0.07010	0.01177
					0.21666	0.00393	0.24826	0.16266	0.17449	0.00524	0.28384	0.17697
				III	1.51164	0.01922	0.07291	0.01148	1.49763	0.01640	0.06653	0.01077
					0.21586	0.00374	0.23947	0.15619	0.18138	0.00514	0.28047	0.17350
			45	I	1.51019	0.02027	0.07529	0.01200	1.48076	0.01728	0.06912	0.01125
			45		0.21642	0.00372	0.24067	0.15798	0.17127	0.00523	0.28429	0.17671
				II	1.51018	0.01693	0.06818	0.01010	1.48194	0.01505	0.06404	0.01001
					0.21462	0.00327	0.22335	0.14577	0.17236	0.00497	0.27479	0.16941
				III	1.50822	0.01656	0.06806	0.00987	1.49334	0.01430	0.06195	0.00952
					0.21470	0.00319	0.22292	0.14549	0.17905	0.00474	0.26336	0.16266
			60	−−	1.50503	0.01562	0.06583	0.00925	1.48008	0.01354	0.06086	0.00913
			60		0.21199	0.00287	0.21081	0.13832	0.17163	0.00472	0.26401	0.16244
	3	40	20	I	1.50019	0.01744	0.06979	0.01076	1.49627	0.01484	0.06316	0.01029
		40	20		0.21572	0.00408	0.25050	0.16015	0.16160	0.00575	0.30320	0.18318
		40	20	II	1.49748	0.01228	0.05844	0.00778	1.49558	0.01157	0.05601	0.00841
					0.21360	0.00328	0.22342	0.14093	0.16736	0.00525	0.28359	0.16980
				III	1.4987	0.01120	0.05564	0.00720	1.50436	0.00994	0.05191	0.00728
					0.2133	0.00319	0.22129	0.13846	0.17736	0.00461	0.26034	0.15613
			30	I	1.49569	0.01219	0.05919	0.00766	1.49418	0.01038	0.05378	0.00773
			30		0.21293	0.00313	0.22087	0.14003	0.16353	0.00509	0.27838	0.16608
			30	II	1.49822	0.01053	0.05469	0.00672	1.4964	0.00948	0.05092	0.00708
					0.21248	0.00290	0.21068	0.13269	0.16804	0.00467	0.2659	0.15841
				III	1.49773	0.01023	0.05395	0.00649	1.50444	0.00910	0.05000	0.00671
					0.21282	0.00275	0.20591	0.12993	0.17605	0.00432	0.24853	0.14926
			40	−−	1.49504	0.01006	0.05334	0.00631	1.49543	0.00879	0.04926	0.00665
			40		0.21165	0.00255	0.19737	0.12535	0.16808	0.00452	0.25882	0.15404
			40									

**Table 2:** LSEs and WLEs of η and μ with their MSEs, RABs, AMSE and ARAB based on 5000 simulations. Population parameter values are η=1.5 and μ=0.2.

		$m_{1}$	$r_{1}$		LSE				WLSE			
		⋮	⋮		$\bar{\hat{\eta }}$	MSE($\hat{\eta }$)	RAB($\hat{\eta }$)	AMSE	$\bar{\overset{ˇ}{\eta }}$	MSE($\overset{ˇ}{\eta }$)	RAB($\overset{ˇ}{\eta }$)	AMSE
M	*ℓ*	$m_{\ell }$	$r_{\ell }$	CS	$\bar{\hat{\mu }}$	MSE($\hat{\mu }$)	RAB($\hat{\mu }$)	ARAB	$\bar{\overset{ˇ}{\mu }}$	MSE($\overset{ˇ}{\mu }$)	RAB($\overset{ˇ}{\mu }$)	ARAB
60	2	30	15	I	1.53847	0.06934	0.13439	0.04335	1.53427	0.06331	0.12874	0.03884
		30	15		0.21641	0.01736	0.50731	0.32085	0.21840	0.01438	0.45715	0.29294
				II	1.51732	0.05361	0.12044	0.03249	1.52090	0.04666	0.11150	0.02780
					0.20097	0.01137	0.41966	0.27005	0.20462	0.00895	0.36927	0.24038
				III	1.51425	0.0442	0.10963	0.02708	1.50998	0.04372	0.10925	0.02647
					0.20677	0.00995	0.3902	0.24992	0.20583	0.00921	0.37535	0.24230
			22	I	1.51872	0.04514	0.11151	0.02837	1.5190	0.04271	0.10857	0.02636
			22		0.20633	0.01159	0.42440	0.26795	0.2079	0.01000	0.39299	0.25078
				II	1.51972	0.04161	0.10676	0.02550	1.52121	0.04005	0.10449	0.02426
					0.20480	0.00940	0.38083	0.24380	0.20696	0.00848	0.35906	0.23178
				III	1.50950	0.03620	0.09975	0.02273	1.50629	0.03527	0.09861	0.02178
					0.20400	0.00926	0.37892	0.23933	0.20386	0.00829	0.35898	0.22880
			30	−−	1.50890	0.03486	0.09826	0.02191	1.51050	0.03348	0.09626	0.02068
			30		0.20234	0.00896	0.37656	0.23741	0.20341	0.00788	0.35227	0.22426
	3	20	10	I	1.52104	0.04246	0.10806	0.02890	1.50653	0.03738	0.10193	0.02512
		20	10		0.20537	0.01533	0.47209	0.29008	0.20908	0.01286	0.42851	0.26522
		20	10	II	1.51030	0.03455	0.09766	0.02251	1.49004	0.02921	0.09036	0.01872
					0.19596	0.01046	0.39092	0.24429	0.19404	0.00822	0.34719	0.21878
				III	1.48314	0.02389	0.08204	0.01606	1.47306	0.02442	0.08327	0.01615
					0.19524	0.00822	0.36154	0.22179	0.19357	0.00788	0.35240	0.21783
			15	I	1.50977	0.02822	0.08836	0.01917	1.50605	0.02678	0.08647	0.01791
			15		0.19966	0.01012	0.39540	0.24188	0.20258	0.00904	0.37310	0.22979
			15	II	1.50582	0.02343	0.08074	0.01564	1.49757	0.02276	0.07976	0.01501
					0.19376	0.00784	0.35263	0.21669	0.19517	0.00726	0.33572	0.20774
				III	1.49938	0.02156	0.07803	0.01460	1.49095	0.02106	0.07719	0.01403
					0.19593	0.00764	0.34721	0.21262	0.19556	0.00701	0.33306	0.20513
			20	−−	1.50700	0.02206	0.07825	0.01481	1.50702	0.02142	0.07711	0.01415
			20		0.19666	0.00756	0.34261	0.21043	0.19928	0.00687	0.32573	0.20142
			20									
120	2	60	30	I	1.51221	0.03432	0.09742	0.02172	1.51203	0.03227	0.09424	0.01989
		60	30		0.20471	0.00912	0.37327	0.23535	0.20724	0.00751	0.33737	0.21581
				II	1.50383	0.02803	0.08824	0.01702	1.50708	0.02329	0.08049	0.01389
					0.19879	0.00602	0.30803	0.19813	0.20184	0.00449	0.26468	0.17259
				III	1.50401	0.02067	0.07548	0.01277	1.50113	0.02053	0.07543	0.01249
					0.20108	0.00487	0.27445	0.17497	0.20094	0.00446	0.26372	0.16958
			45	I	1.50642	0.02376	0.08115	0.01496	1.50792	0.02239	0.07868	0.01380
			45		0.20144	0.00617	0.30848	0.19481	0.20340	0.00522	0.28293	0.18080
				II	1.50260	0.01915	0.07324	0.01181	1.50436	0.01848	0.07197	0.01118
					0.19922	0.00447	0.26620	0.16972	0.20141	0.00389	0.24736	0.15967
				III	1.50569	0.01812	0.07102	0.01141	1.50349	0.01764	0.07005	0.01091
					0.20304	0.00469	0.27226	0.17164	0.20301	0.00417	0.25616	0.16310
			60	−−	1.50372	0.01729	0.06921	0.01083	1.50533	0.01641	0.06756	0.01007
			60		0.19946	0.00437	0.26068	0.16495	0.20098	0.00373	0.24079	0.15418
	3	40	20	I	1.50804	0.02143	0.07695	0.01448	1.50185	0.01968	0.07387	0.01306
		40	20		0.19946	0.00754	0.34122	0.20908	0.20384	0.00644	0.31336	0.19362
		40	20	II	1.50454	0.01655	0.06833	0.01085	1.49144	0.01384	0.06264	0.00881
					0.19540	0.00515	0.28233	0.17533	0.19518	0.00378	0.24437	0.15350
				III	1.49479	0.01226	0.05832	0.00818	1.48801	0.01245	0.05902	0.00814
					0.19821	0.00411	0.25513	0.15673	0.19759	0.00383	0.24634	0.15268
			30	I	1.50478	0.01463	0.06361	0.00989	1.50278	0.01389	0.06186	0.00919
			30		0.19803	0.00516	0.28418	0.17390	0.20082	0.00449	0.26447	0.16316
			30	II	1.50127	0.012	0.05802	0.00791	1.49621	0.01153	0.05699	0.00746
					0.19636	0.00382	0.24638	0.1522	0.19791	0.00339	0.2318	0.14439
				III	1.49731	0.01125	0.05630	0.00757	1.49163	0.01099	0.05566	0.00724
					0.19674	0.00388	0.24655	0.15142	0.19681	0.00348	0.23349	0.14457
			40	−−	1.50099	0.01069	0.05489	0.00723	1.50081	0.01036	0.05405	0.00684
			40		0.19907	0.00376	0.24161	0.14825	0.20116	0.00331	0.22620	0.14013
			40									

**Table 3:** AILs and COVP (in %) of 95% CIs of η and μ based on 5000 simulations. Population parameter values are η=1.5 and μ=0.2.

		$m_{1}$	$r_{1}$		NACI			LTCI		
		⋮	⋮		CI(η)	AIL(η)	COVP(η)	CI(η)	AIL(η)	COVP(η)
M	*ℓ*	$m_{h}$	$r_{h}$	CS	CI(μ)	AIL(μ)	COVP(μ)	CI(μ)	AIL(μ)	COVP(μ)
60	2	30	15	I	(1.0616,1.9988)	0.9372	95.38	(1.1268,2.0788)	0.9521	96.04
		30	15		(0.0512,0.4167)	0.3655	96.50	(0.1017,0.6088)	0.5071	91.40
				II	(1.1249, 1.9470)	0.8221	95.52	(1.1754, 2.0074)	0.8320	95.02
					(0.0631, 0.3969)	0.3337	94.92	(0.1090, 0.5386)	0.4296	90.70
				III	(1.1427, 1.9144)	0.7717	95.22	(1.1876, 1.9676)	0.7800	94.80
					(0.0652, 0.3898)	0.3246	95.12	(0.1095, 0.5157)	0.4062	90.88
			22	I	(1.1251, 1.9179)	0.7928	95.02	(1.1726, 1.9745)	0.8018	94.94
			22		(0.0627, 0.3879)	0.3252	95.28	(0.1076, 0.5202)	0.4126	91.58
				II	(1.1532, 1.8920)	0.7389	95.26	(1.1946, 1.9408)	0.7462	95.30
					(0.0699, 0.3803)	0.3103	95.52	(0.1119, 0.4848)	0.3729	91.66
				III	(1.1632, 1.8811)	0.7179	94.82	(1.2025, 1.9270)	0.7246	95.04
					(0.0697, 0.3757)	0.3060	95.70	(0.1111, 0.4778)	0.3667	91.96
			30	−−	(1.1722, 1.8614)	0.6892	94.42	(1.2086, 1.9037)	0.6952	94.28
			30		(0.0745, 0.3670)	0.2925	94.64	(0.1135, 0.4585)	0.3450	91.52
	3	20	10	I	(1.1476, 1.8597)	0.7121	94.74	(1.1867, 1.9055)	0.7188	95.52
		20	10		(0.0591, 0.3970)	0.3379	95.76	(0.1057, 0.5599)	0.4542	91.40
		20	10	II	(1.1932, 1.8209)	0.6277	94.96	(1.2237, 1.8560)	0.6322	95.34
					(0.0682, 0.3785)	0.3103	95.04	(0.1105, 0.4733)	0.3628	91.18
				III	(1.2102, 1.7977)	0.5876	94.18	(1.2371, 1.8284)	0.5913	94.96
					(0.0730, 0.3739)	0.3009	94.94	(0.1133, 0.4640)	0.3507	90.60
			15	I	(1.1930, 1.8046)	0.6116	94.28	(1.2222, 1.8381)	0.6159	94.80
			15		(0.0708, 0.3710)	0.3002	95.38	(0.1112, 0.4604)	0.3492	91.94
			15	II	(1.2145, 1.7838)	0.5693	94.06	(1.2400, 1.8127)	0.5727	94.46
					(0.0758, 0.3636)	0.2878	95.32	(0.1141, 0.4402)	0.3261	92.26
				III	(1.2197, 1.7756)	0.5558	94.10	(1.2440, 1.8030)	0.5590	94.64
					(0.0776, 0.3622)	0.2846	95.30	(0.1152, 0.4347)	0.3195	91.70
			20	−−	(1.2241, 1.7692)	0.5451	94.90	(1.2475, 1.7957)	0.5481	95.36
			20		(0.0815, 0.3552)	0.2737	95.70	(0.1170, 0.4196)	0.3026	91.94
			20							
120	2	60	30	I	(1.1820, 1.8457)	0.6637	95.70	(1.2159, 1.8850)	0.6690	95.52
		60	30		(0.0835, 0.3554)	0.2719	95.88	(0.1186, 0.4170)	0.2984	93.08
				II	(1.2295, 1.8033)	0.5738	95.78	(1.2550, 1.8322)	0.5773	95.58
					(0.0966, 0.3373)	0.2407	95.42	(0.1253, 0.3826)	0.2573	92.22
				III	(1.2429, 1.7804)	0.5376	95.22	(1.2654, 1.8058)	0.5404	94.98
					(0.0992, 0.3329)	0.2337	94.96	(0.1266, 0.3752)	0.2486	92.24
			45	I	(1.2339, 1.7864)	0.5525	95.06	(1.2577, 1.8134)	0.5556	95.12
			45		(0.0996, 0.3336)	0.2340	95.24	(0.1270, 0.3756)	0.2486	92.10
				II	(1.2534, 1.7669)	0.5135	95.08	(1.2741, 1.7901)	0.5160	95.12
					(0.1043, 0.3252)	0.2209	95.26	(0.1291, 0.3622)	0.2331	92.88
				III	(1.2584, 1.758)	0.4996	94.96	(1.2780, 1.7799)	0.5019	94.86
					(0.1055, 0.324)	0.2185	95.40	(0.1298, 0.3599)	0.2301	92.46
			60	−−	(1.2638, 1.7462)	0.4824	94.58	(1.2822, 1.7667)	0.4845	94.88
			60		(0.1079, 0.3162)	0.2084	95.00	(0.1304, 0.3488)	0.2185	93.08
	3	40	20	I	(1.2423, 1.7581)	0.5158	94.32	(1.2633, 1.7816)	0.5184	94.52
		40	20		(0.0909, 0.3413)	0.2504	95.68	(0.1218, 0.3919)	0.2701	92.94
		40	20	II	(1.2782, 1.7168)	0.4386	94.68	(1.2935, 1.7337)	0.4402	94.68
					(0.1028, 0.3246)	0.2219	95.06	(0.1279, 0.3621)	0.2342	92.52
				III	(1.2923, 1.7051)	0.4129	94.62	(1.3058, 1.7200)	0.4142	94.48
					(0.1060, 0.3208)	0.2148	95.06	(0.1297, 0.3556)	0.2259	92.46
			30	I	(1.2779, 1.7135)	0.4356	94.72	(1.2930, 1.7302)	0.4371	95.20
			30		(0.1046, 0.3214)	0.2168	95.46	(0.1287, 0.3568)	0.2281	93.14
			30	II	(1.2969, 1.6995)	0.4027	94.84	(1.3098, 1.7137)	0.4039	95.2
					(0.1101, 0.3149)	0.2048	94.66	(0.1319, 0.3462)	0.2143	92.94
				III	(1.3014, 1.6941)	0.3927	95.04	(1.3137, 1.7075)	0.3938	95.50
					(0.1116, 0.3141)	0.2025	94.98	(0.1329, 0.3443)	0.2114	92.98
			40	−−	(1.3026, 1.6875)	0.3849	94.46	(1.3145, 1.7004)	0.3860	94.64
			40		(0.1145, 0.3088)	0.1943	94.90	(0.1343, 0.3365)	0.2022	93.50
			40							

**Table 4:** The competing continuous models of the KMILBE distribution with their pdfs and cdfs.

Models	Abbreviation	PDF	CDF
Inverse length biased exponential	ILBE	$f\left(x\right)=\theta^{2}x^{-3}e^{-\frac{\theta }{x}}$	$F\left(x\right)=\left(1+\frac{\theta }{x}\right)e^{-\frac{\theta }{x}}$
Sine inverse exponential	SIE	$f\left(x\right)=\frac{\pi \theta }{2x^{2}}e^{-\frac{\theta }{x}}cos\left[\frac{\pi }{2}e^{-\frac{\theta }{x}}\right]$	$F\left(x\right)=sin\left[\frac{\pi }{2}e^{-\frac{\theta }{x}}\right]$
Sine inverse Rayleigh	SIR	$f\left(x\right)=\frac{\pi \theta }{2x^{3}}e^{-\frac{\theta }{x^{2}}}cos\left[\frac{\pi }{2}e^{-\frac{\theta }{x^{2}}}\right]$	$F\left(x\right)=sin\left[\frac{\pi }{2}e^{-\frac{\theta }{x^{2}}}\right]$
Inverse Lindley	IL	$f\left(x\right)=\frac{\theta^{2}}{1+\theta }\left(\frac{1+x}{x^{3}}\right)e^{\frac{-\theta }{x}}$	$F\left(x\right)=\left(1+\frac{\theta }{(1+\theta )x}\right)e^{\frac{-\theta }{x}}$
Lindley	L	$f\left(x\right)=\frac{\theta^{2}}{1+\theta }\left(1+x\right)e^{-\theta x}$	$F\left(x\right)=1-\left(1+\frac{\theta x}{1+\theta }\right)e^{-\theta x}$
Inverse exponential	IE	$f\left(x\right)=\theta x^{-2}e^{-\frac{\theta }{x}}$	$F\left(x\right)=e^{-\frac{\theta }{x}}$

**Table 5:** The goodness of fit tests for data set 1.

Models	-LL	AIC	CAIC	BIC	HQIC	KS	PV	MLE and SE
KMILBE(θ)	357.423	716.845	716.956	716.425	717.428	0.1444	0.407	10,190 (1048.837)
ILBE(θ)	358.278	718.556	718.667	718.136	719.139	0.1715	0.213	8414 (965.099)
SIE(θ)	359.098	720.196	720.307	719.776	720.779	0.1848	0.1491	5602 (696.008)
SIR(θ)	362.625	727.251	727.362	726.831	727.834	0.2182	0.0536	4389 (270.107)
IE(θ)	367.001	736.002	736.336	735.582	736.585	0.3031	0.0019	4207 (682.428)
IL(θ)	367.001	736.002	736.336	735.582	736.585	0.3031	0.0019	4208 (682.428)

**Table 6:** The goodness of fit tests for data set 2.

Models	-LL	AIC	CAIC	BIC	HQIC	KS	PV	MLE and SE
KMILBE(θ)	−2.205	−2.411	−2.257	−2.964	−2.003	0.1375	0.665	0.562 (0.069)
SIR(θ)	10.921	23.842	23.996	23.289	24.249	0.30611	0.0105	0.237 (0.017)
IE(θ)	1.248	4.496	4.958	3.943	4.903	0.2279	0.1091	0.237 (0.045)
IL(θ)	−1.167	−0.334	−0.181	−0.887	0.073	0.1554	0.5084	0.406 (0.055)
L(θ)	0.294	2.588	2.742	2.742	2.996	0.18995	0.2645	3.27 (0.520)

## Data Availability

Data sets are available in the application section.
